# A framework for impact based heat stress warning system for a coastal city in India

**DOI:** 10.1038/s41598-026-38639-9

**Published:** 2026-03-06

**Authors:** Kshitij Kacker, Abhinav Utpal, Shiwam Singh, Piyush Srivastava, Mahua Mukherjee, Srikrishnan Siva Subramanian, R. Rakesh

**Affiliations:** 1 Department of Architecture and Planning, Indian Institute of Technology Roorkee, Roorkee, Uttarakhand 247667 India; 2 Centre of Excellence in Disaster Mitigation and Management, Indian Institute of Technology Roorkee, Roorkee, Uttarakhand 247667 India; 3 Tarutium Global Consulting, Chennai, India

**Keywords:** Climate sciences, Environmental sciences

## Abstract

Urban Heat Islands (UHIs) pose a significant challenge to densely populated cities of India, intensifying heat stress and impacting public health, energy consumption, and urban sustainability. This study aims to identify Urban Heat Stress Risk in Mumbai (India) through the heat stress event of April-May 2024 using satellite data and by assessing thermal stress risk through a systematic approach combining urban weather modelling and in-situ observations along with vulnerability and exposure of the residents. Satellite-derived surface temperature data is analyzed to detect spatial patterns of UHI hotspots across the region. To deepen the understanding of thermal dynamics, the Weather Research and Forecasting Model with Urban Canopy Model (WRF-UCM) is employed, utilizing Local Climate Zone (LCZ) global data to simulate the Universal Thermal Climate Index (UTCI). A nested domain structure with spatial resolutions of 3*3 km, 1*1 km and 333*333 m are used to capture regional and localized variations of urban weather parameters. Validation of the WRF-UCM simulated meteorological variables is carried out using data from 36 Automatic Weather Stations across Mumbai (India). A correlation analysis between satellite-derived UHI hotspots and validated model results provides comprehensive insights into heat stress distribution. The findings highlight critical zones experiencing increased heat stress. The vulnerability and exposure index formulated through the Principal Component Analysis tool helped as additional overlaid layers to assess the risk over the study area, serving as an additional tool for urban planning interventions and policy recommendations related to heat stress risk.

**Supplementary Information:**

The online version contains supplementary material available at 10.1038/s41598-026-38639-9.

## Introduction

Urbanization has transformed the landscape of the cities worldwide at a very rapid pace, leading to sprawling developments dominated by the concrete, asphalt and impervious surfaces. This transformation not only alters natural environment but also affects the surface energy budget, leading to significant changes in the temperature of urban and rural areas which in common terms, referred as the Urban Heat Island (UHI) effect^1–3^. Cities which were used to be balanced by the green areas and open spaces are now facing the phenomenon of localized warming, which results in the heightened heat stress, reduced thermal comfort and increased energy demands for cooling during the summer months. Additionally, climate change is a complex socio-ecological issue which is mainly enhanced by anthropogenic activities and is impacting the cities by changing air temperature and patterns of precipitation, thereby increasing the frequency of heatwave events^2,4,5^.Heat stress is one of such hazards which results from a combination of environmental factors (such as the air temperature, humidity, radiation, velocity), clothing requirements and metabolic heat production. Specifically, the elderly population, children and people who are involved in enormous physical labour activities in exposed environment (such as construction labours) are at higher risks^6^. In India 2% of the work capacity is already affected by the severe heat waves which is likely to see an increase of 8% by the end of 21st centuy causing a reduction in the work performance by 30–40%^7,8^. The situation highlighs the urgent need to adapt to climate change, as there is high risk to economic and health issues that the country may suffer due to the heat stress^9,10^.

UHI effect, increasing vulnerable population, rapid growth and frequent heatwaves encounters are the reasons due to which the urbanized areas are the most affected^11–13^. Currently, with 11% of the urban dwellers of world are living in the Indian cities making India the second largest urban system. An improved assessment of risk is required, considering the fact that, accelerated urbanization will increase exposed population to extreme thermal conditions^14^.

In this study, the combination of Universal Thermal Climate Index (UTCI) which is a human thermal comfort model developed through the Numerical Weather Prediction (NWP) Models, along with vulnerability and exposure indices, was used to evaluate the heat stress risk at ward level for coastal city. This framework, originally proposed by Kacker et al. (2024), can be used to study the heat stress risk distribution at the ward level by the policymakers and planners for effectively planning the strategies and implementing them as per the necessity and priority^15^. This study utilises the method to assess the heat stress risk distribution at the ward level for the coastal city for the April-May 2024 heat scenario.

## Data and methodology

### Study area and data set

**Fig. 1:**
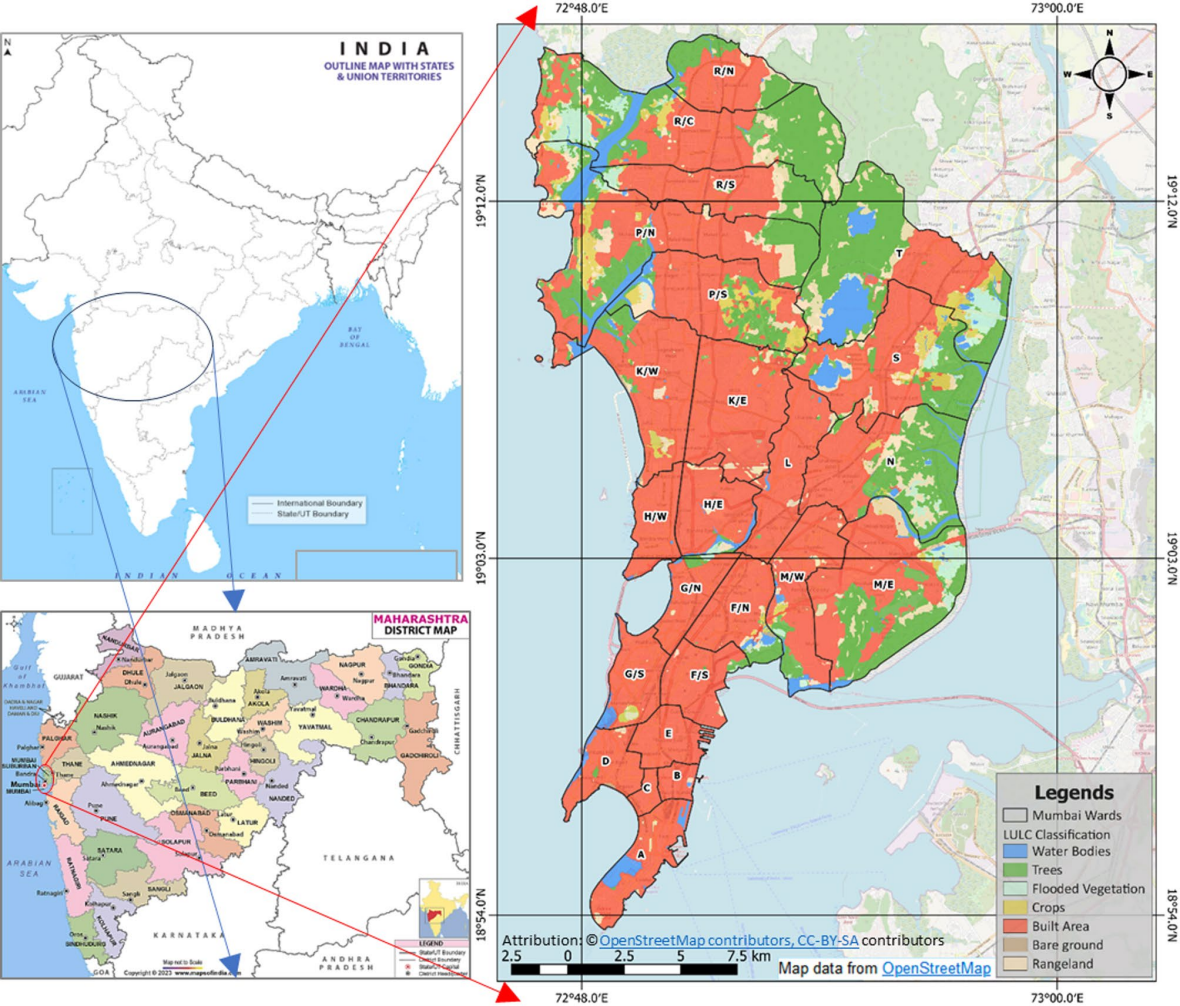
Study area (Mumbai) and its location within India.

The study was conducted on the Municipal Corporation of Greater Mumbai (MCGM) boundary formally known as Bombay Municipal Corporation (BMC). Mumbai, being the 5th largest city in the world, is costal city also referred as finance capital of India. MCGM consists of the Mumbai Island city and the Mumbai Suburban Districts as shown in Fig. 1. The area is located at an average altitude of 14 m above the mean sea level. According to the 2011 census, the MCGM consists of 24 wards covering and area of 437.71 Sq. Km., in which 9 wards (68.71 Sq. Km.) are in the Mumbai Island City and the rest 15 (369 Sq. Km.) are from the Mumbai Suburban Districts. According to the BMC (2021) MCGM had a 12.4 million population residing in the urban area. As per the Köppen climate classification the MCGM area lies in tropical wet and dry climate. The region experiences typically two main season – the humid season and the dry season, usually the period of from October to May is relatively dry. As per Mumbai Metropolitan Regional Development Authority (MMRDA), it is projected that by 2036, approximately 5 million new residential units will be required to fulfil Mumbai’s future demand^16^. The city covers a length of 274.1 km as coastline, with high relative humidity in the region, ranging from 54% to 85%^17^. In terms of growth pattern, the Mumbai Metropolitan Region (MMR) has shown a growth of 89% over two decades, where peripheral zones near the cores have exhibit high growth rates, leading to a diverse growth pattern^18^. Hence, due a long coastline along with high urbanisation rate, the study depicts novelty to assess the impact of UTCI-based heat stress risk in urban area of MMR.The Land Surface Temperature (LST) for the Municipal Corporation of Greater Mumbai (MCGM) was derived using Landsat 8 and Landsat 9 Level 2 Collection 2 data from the USGS Earth Explorer platform for the dates 23rd April 2024, 01st May 2024 and 09th May 2024, to match the simulation dates, which were further averages to formulate the LST conditions. Further, in-situ data from 54 Automatic Weather Stations within the Municipal Corporation of Greater Mumbai (MCGM) were utilized for this study. After data cleaning and sorting process, 36 stations were found containing the data for validation purpose.

### Model setup

The variable required for the estimation of outdoor heat stress hazard was simulated using the Weather Research and Forecast (WRF) model^19^ combining with the Building Effect Parameterization (BEP) Urban Canopy Model (WRF-UCM)^20^. Global Local Climate Zone (LCZ) data having a 100 m resolution was used for integrating the morphological characteristics^21^. “Local Climate Zones (LCZs) are a region of uniform surface cover, material, structure and human activity, that spans over 100s of metres to kilometres in horizontal scale”^22^. Hence, they encompass the morphology of the city, anthropogenic heat release and a synoptic view of the city’s structure and fabric to provide a unique identity to a region^23,24^. The WRF-UCM model provides output variable like air velocity, air temperature, humidity and values related to radiation, which ware used to compute the UTCI – a widely recognized thermal stress indicator^25^. The UTCI map, representing the heat stress intensity, served as the hazard component.

**Fig. 2:**
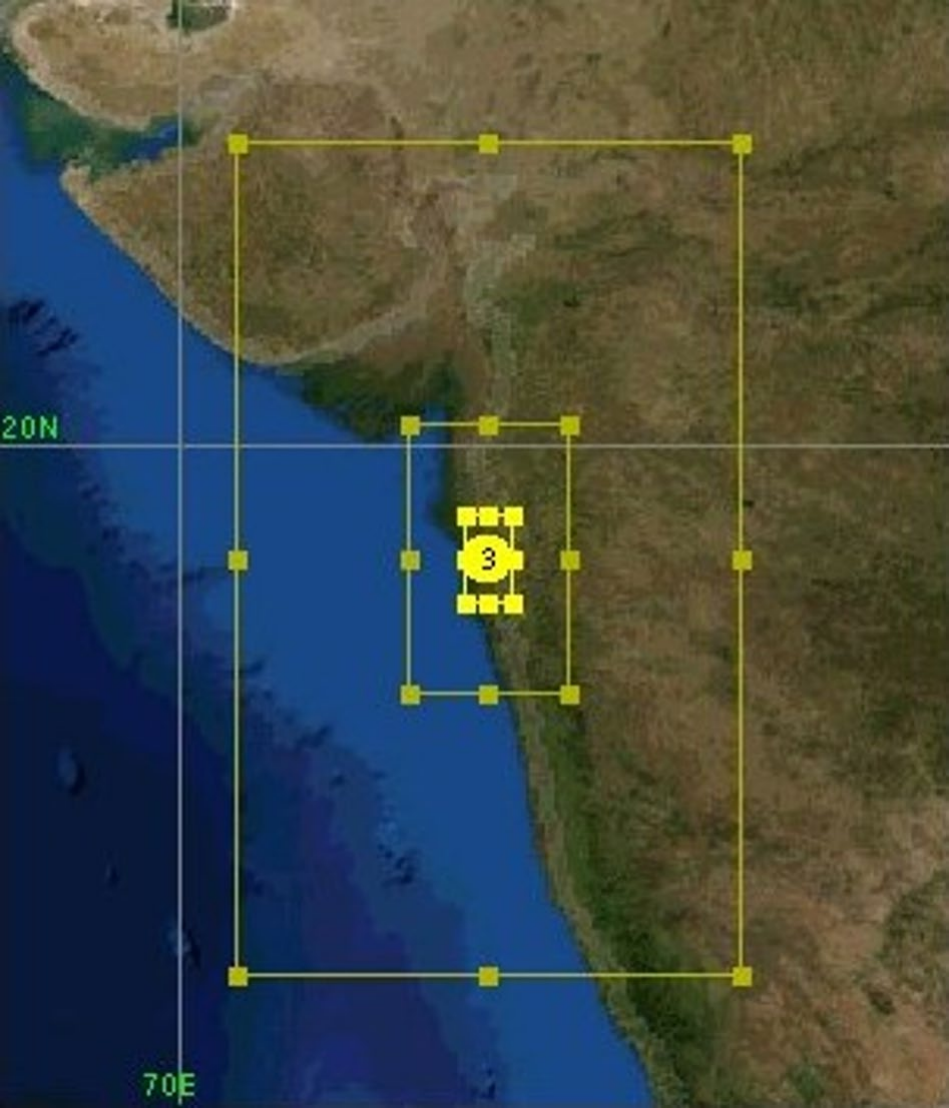
The three nested domains, namely, domain D01 (3 km × 3 km), domain D02 (1 km × 1\ km) and domain D03 (0.33 km × 0.33 km), used for the WRF model.

The model simulation setup consists of three nested domains, with the highest resolution reaching 333 m. The outermost domain (D01) covers a large portion of the Indian subcontinent at a 3 km resolution. The second domain (D02) is the first nested domain focuses on South-western India and follows a 1:3 nesting ratio leading to a higher resolution of 1 km. The third domain (D03) is nested within D02 at a 1:3 ratio covering the municipal boundary of MCGM with a fine resolution of 333 m. All three domains in this study are centred at 19.0°N and 72.857°E, as illustrated in Fig. 2. The WRF-UCM BEP is a multi-layered urban model within the WRF framework that provides a more detailed representation of urban environments^26^. The global LCZ map was developed using a random forest classifier at a 100 m spatial resolution to categorize Earth’s surface into 17 local climate zone classes based on observational imagery^21^. Using this LCZ dataset and the w2w tool^27^, geographic input files were generated to incorporate urban area details for the study domains used in the WRF-UCM simulations.

In similar studies the simulation setup was distributed into smaller segments. Mohan & Sati, (2016) in their study explained that smaller split simulation ranging from 2 to 4 days show better performance than in comparison to longer continuous runs^28^. Mohan, et al., (2020) in their work, considered a run for 4 days under which 12 h are used as model spin up and rest 84 h are used for analysis^29^; Srivastava, et al., (2021), considered a run for 4 days under which 24 h are used as model spin up^30^. Similarly, to assess an event Srivastava, et al., (2021) performed a 12-day simulation^30^; Kacker, et al., (2024) performed a 5-day simulation^15^; Singh, et al., (2022) performed a 16-day simulation^31^. Hence, to understand the heat stress conditions a 2.5 weeks time was considered which was divided into simulation of 3 day runs consisting of total 5 runs. This helped in to capture the extreme heat event and to average out the result that helped in reducing any data anomaly. If the whole summer period would have been considered then it would might have substantially decreased the peak average value that would have affected the heat stress risk values. Additionally, the first 24 h of each simulation were designated as spin-up time and discarded. The physical parameterization schemes used in the simulation follow^15^ and summarized in Table 1.

**Table 1:** Summary of WRF-UCM model setup.

Parameter	Description of Data	Reference
Date Selection	NCEP - FNL	^32^
Duration of Simulation	23/04/2024-09/05/2024	
Temporal interval of boundary data	6 h	
Domain Resolution	Domain 1–3 km	
	Domain 2 –1 km	
	Domain 3–0.33 km	
Microphysics	Purdue Lin scheme	^33^
Longwave radiation	Rapid Radiative Transfer Model (RRTM) scheme	^34^
Shortwave radiation	Dudhia scheme	^35^
Surface layer scheme	Revised MM5 Monin-Obukhov scheme	^36^
Land surface model	Noah land surface model	^37^
Cumulus parameterization	Kain–Fritsch cumulus parameterization	^38^

### Heat risk index

#### Hazard (UTCI) variable

The UTCI is widely recognized for its ability to provide a standardized measure of thermal stress by integrating multiple environmental variables. It is based on an advanced multi-node thermoregulation model, making it more reliable than single-variable indices. Unlike basic single-parameter indices, the UTCI considers air temperature ($\:{T}_{a}$), humidity (expressed as vapor pressure, $\:{V}_{p}$), wind speed ($\:{v}_{a}$), and radiation (measured through mean radiant temperature, $\:{T}_{mrt}$), allowing for a more comprehensive evaluation of human thermal comfort^39^. The UTCI is specifically designed to analyze outdoor heat stress conditions^25^ and is used to understand the heat stress within urban areas^24,40^. By utilizing the novelty brought by^15^ to use the WRF-UCM model output to calculate the MRT at the city level, UTCI is then formed using the required inputs in polynomial equation produced and shared under COST Action 730^41,42^ (detailed in Supplementary_Material Appendix A and B). The UTCI map, generated at a 333-meter resolution, was aggregated for each of the 24 municipal wards in MCGM to obtain ward-wise average UTCI values. UTCI values are categorized into 10 stress levels, covering a full spectrum of thermal stress conditions: 5 categories for cold stress, 1 category for neutral (no) stress, and 4 categories representing heat stress conditions. To create a heat stress hazard index, the four heat stress categories were re-indexed on a common scale of 0 to 1, aligning them with vulnerability and exposure indices (see Table 2). Additionally, values within each heat stress category were further normalized to form a heat index for each ward in MCGM.

**Table 2:** Normalized UTCI heat stress according to category ranging from 0 to 1.

UTCI (^◦^C) range	Stress category	Normalized range
Above + 46	Extreme heat stress	1.00
+ 38 to + 46	Very strong heat stress	0.75 to 1.00
+ 32 to + 48	Strong heat stress	0.50 to 0.75
+ 26 to + 32	Moderate heat stress	0.25 to 0.50
+ 9 to + 26	No thermal stress	0 to 0.25

The meteorological parameters used for UTCI calculation (air temperature, relative humidity, wind speed, and solar radiation) were validated against data from automatic weather stations. The model’s performance was assessed using statistical metrics, including: Pearson Correlation Coefficient (PCC), Root Mean Square Error (RMSE), Mean Absolute Error (MAE), Index of Agreement (IoA)^43–46^. Further details on the mathematical models and validation results are provided in Supplementary_Material Appendix C.

#### Vulnerability and exposure variables

Indicators of vulnerability are generally divided into two main categories: biophysical vulnerability, which reflects the sensitivity of the natural environment to hazards, and social vulnerability, which pertains to the sensitivity and adaptability of human populations^47,48^. In this study, the methodology outlined in^15^ has been utilized. For the city of Mumbai, relevant demographic and socioeconomic data were obtained from the Census of India (see Table 3), covering all administrative wards. The vulnerability indicators included population density^11,49–51^, household density^51–53^ and Socially weaker sections ^51,54^. For exposure indicators, the parameters considered were the proportion of the population engaged in marginal work^49,55,56^, illiteracy rates^12,52,57^, prevalence of substandard housing conditions^58–60^, lack of access to electricity^59^, poor household water quality, and distance to primary water sources^50^. Census 2011 remains the most recent comprehensive demographic dataset available in India. It is a few Indian sources which provides population, household data, etc. at the ward level. Cunningham, et al., (2024) used the Census 2011 child labour dataset at the district level for performing Global Regression for the whole India^61^. The 2021 India Census has been postponed until at least 2027^62^ but the format of this study provides a framework that could be applied to and compared with future editions of the dataset. Hence, the Census 2011 data are the latest data collected by the Government of India and are currently being used by researchers.

**Table 3:** Hazard, Vulnerability, and exposure indicators and their functional relationships.

Index	Indicators	Description	Unit	Functional relationship	Source
Hazard	UTCI	Universal Thermal Climate Index in each ward	°C	+ve	WRF_UCM Model
Vulnerability	Population Density	Population per unit square kilometer in each ward	Population /Sq. km.	+ve	Census 2011 (Latest Official Records)
	Household Density	Households per unit square kilometer in each ward	Households /Sq. km.	+ve	Census 2011 (Latest Official Records)
	Social Weaker Section	Percentage of socially weaker sections to total population in each ward	%	+ve	Census 2011 (Latest Official Records)
Exposure	Marginal Work	Percentage of marginal workers to total population in each ward	%	+ve	Census 2011 (Latest Official Records)
	Illiteracy	Percentage of illiterate individuals to total population in each ward	%	+ve	Census 2011 (Latest Official Records)
	Dilapidated Household Condition	Percentage of households in dilapidated condition to total households in ward	%	+ve	Census 2011 (Latest Official Records)
	Household No Electrical Connection	Percentage of households with no electricity connection in each ward	%	+ve	Census 2011 (Latest Official Records)
	Untreated Water Quality Household	Percentage of households with untreated water in each ward	%	+ve	Census 2011 (Latest Official Records)
	Household with Distant Water Source	Percentage of households with distant water sources in each ward	%	+ve	Census 2011 (Latest Official Records)

#### Risk equation

Vulnerability and exposure indicators were normalized using min–max normalization. The equations used are as follows^63^:

For positively correlated indicators:

1 $$\:\begin{array}{c}K=\frac{{Z}_{i}-{Z}_{min}}{{Z}_{max}-{Z}_{min}}\end{array}$$

For negatively correlated indicators:

2 $$\:\begin{array}{c}K=1-\frac{{Z}_{i}-{Z}_{min}}{{Z}_{max}-{Z}_{min}}\end{array}$$

Here, $\:{Z}_{i}$ is the value of the parameter for the ith ward, and $\:{Z}_{min}$ and $\:{Z}_{m\mathrm{a}\mathrm{x}}$ are the minimum and maximum values of that parameter across all wards, respectively.

Similar to^15^, Principal Component Analysis (PCA) is employed separately for the vulnerability and exposure indicators to reduce dimensionality. The first set of PCA includes vulnerability indicators containing 3 parameters, and the second set of PCA is used to extract components for the exposure indicator, which contains 6 parameters. The PCA calculations were performed with the IBM SPSS Statistics 26^64^ tool. Bartlett’s test and Kaiser–Meyer–Olkin (KMO) tests were performed on the data to examine the suitability of the data in terms of the common variance proportion and identity correlation matrix. The PCA technique is helpful as correlated variables get combined to formulate new independent uncorrelated variables or principal components (PCs) that follow a linear combination with the original set of variables^65^. The PCA value above 0.5 has been acceptable for such kind of studies. ^15^ got a KMO of 0.504 for creating Vulnerability Index;^52^ got a KMO of 0.500 while formulating Exposure Index. As per Nardo, et al., (2005) and Hair, et al., (2014) in their studies defined that 0.5 KMO value is sufficient for adopting PCA^66,67^. Subsequently, regression factor scores were computed for each component under both vulnerability and exposure categories. These scores were then used to calculate the overall vulnerability and exposure index for each ward in the city. This was done by summing the product of each weighted factor score, as outlined in previous studies^52,68^:

3 $$\:\begin{array}{c}\mathrm{I}\mathrm{n}\mathrm{d}\mathrm{e}\mathrm{x}=\frac{{\sum\:}_{j=1}^{m}{\sum\:}_{i=1}^{n}{C}_{i}{W}_{j}}{{\sum\:}_{j=1}^{m}{W}_{j}}\end{array}$$

Where $\:{C}_{i}\text{}$ is the normalized component score for the $\:{i}^{\mathrm{th}}$ ward, $\:{W}_{j}$ is the weight assigned to the $\:{j}^{th}$ principal component, n is the total number of wards, m is the number of principal components.

The Heat Stress Risk Index (HSRI) was then calculated using the following formula^69^:

4 $$\:\begin{array}{c}HSRI=HI\times\:VI\times\:EI \end{array}$$

Here, HI is the Hazard Index, VI stands for Vulnerability Index and EI denotes the Exposure Index.

All indices except the hazard index were classified into five categories based on the number of standard deviations (SDs) from the mean, following the method described in^52,70,71^. The classification scheme is as follows Lowest refers to < − 1.5 SD, Low indicates − 1.5 SD to − 0.5 SD, Moderate indicates − 0.5 SD to + 0.5 SD, High indicates + 0.5 SD to + 1.5 SD and Highest emphasize > + 1.5 SD.

## Results

### Land surface temperature

**Fig. 3:**
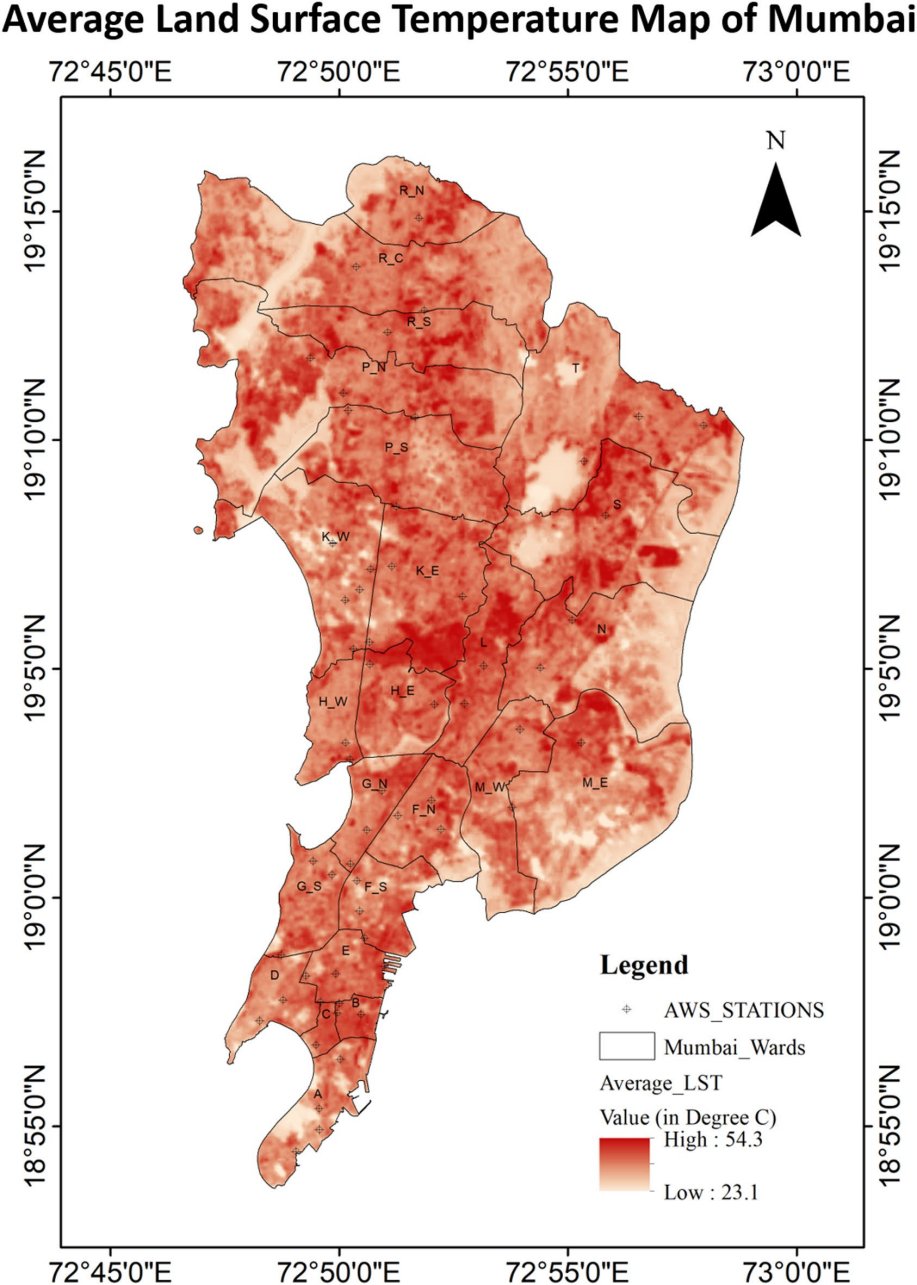
Land surface temperature (LST) map, Mumbai (Summer 2024).

Figure 3 illustrates the spatial distribution of the land surface temperature over the Mumbai Metropolitan Region for summer month of 2024 consisting the average of three different time frame 23th April, 01st May and 09th May of 2024 using the raster calculator in QGIS Software. These dates matched the simulation period and help in assessing the heat event. The LST values were visualized using a colour gradient, where darker orange colour represented higher temperatures. The maximum average LST was observed 54.3 °C and the minimum average temperature was around 23.1 °C during the period. The minimum temperature was observed mainly over the LCZ-G (Water), indicating the cooling effect of water bodies on surrounding areas. On the other hand, the relatively higher temperature was observed primarily in densely built-up regions, such as LCZ-2 (Compact Mid-rise) and LCZ-3 (Compact Low-Rise), which are characterized by a high density of buildings and impervious surfaces in the Mumbai Metropolitan Region. These areas with higher average LST are vulnerable to the urban heat island effect and in need of targeted interventions, such as the introduction of more green spaces or reflective materials in urban planning.

### Model validation

**Fig. 4:**
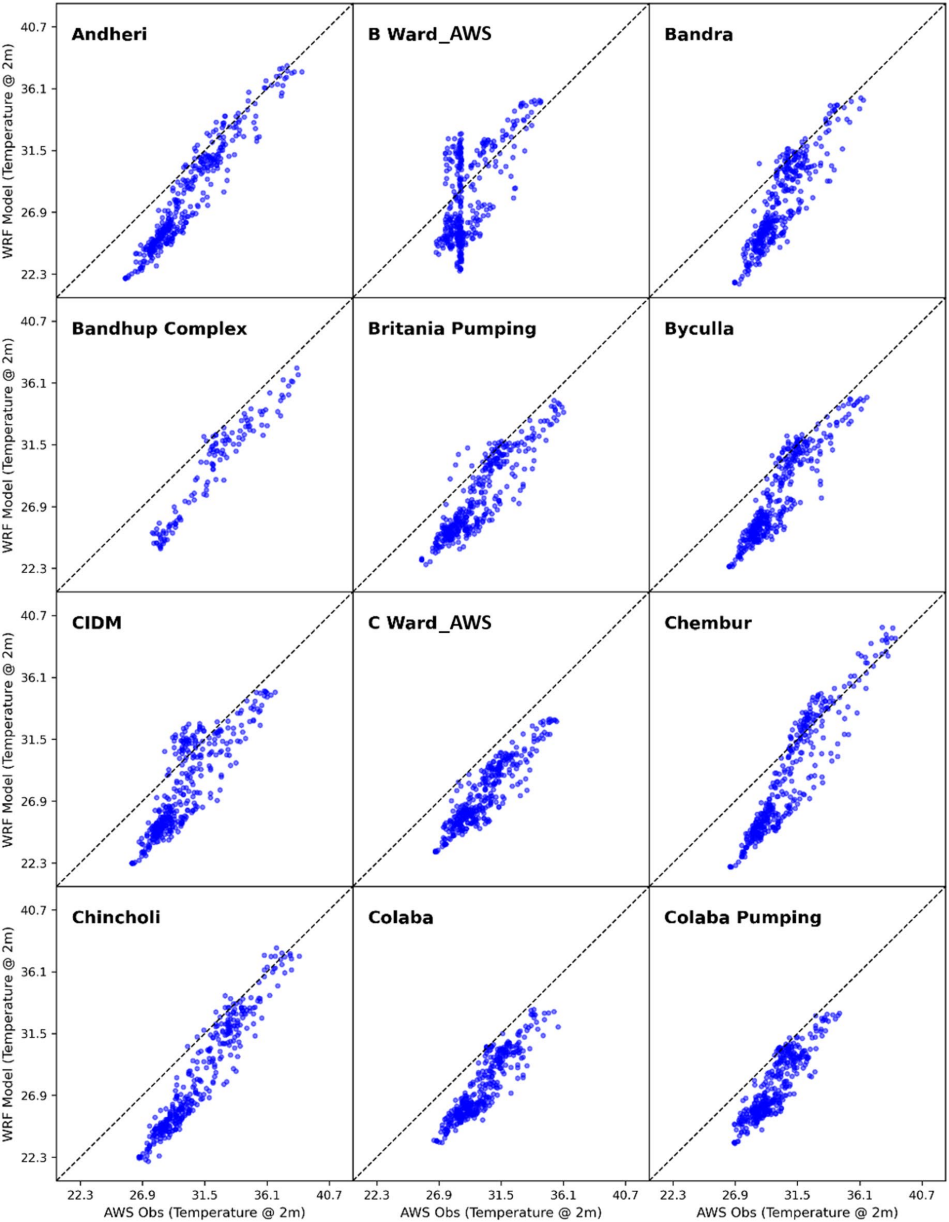
Scatter plot of WRF model simulated vs. AWS observed 2 m temperature.

**Fig. 5:**
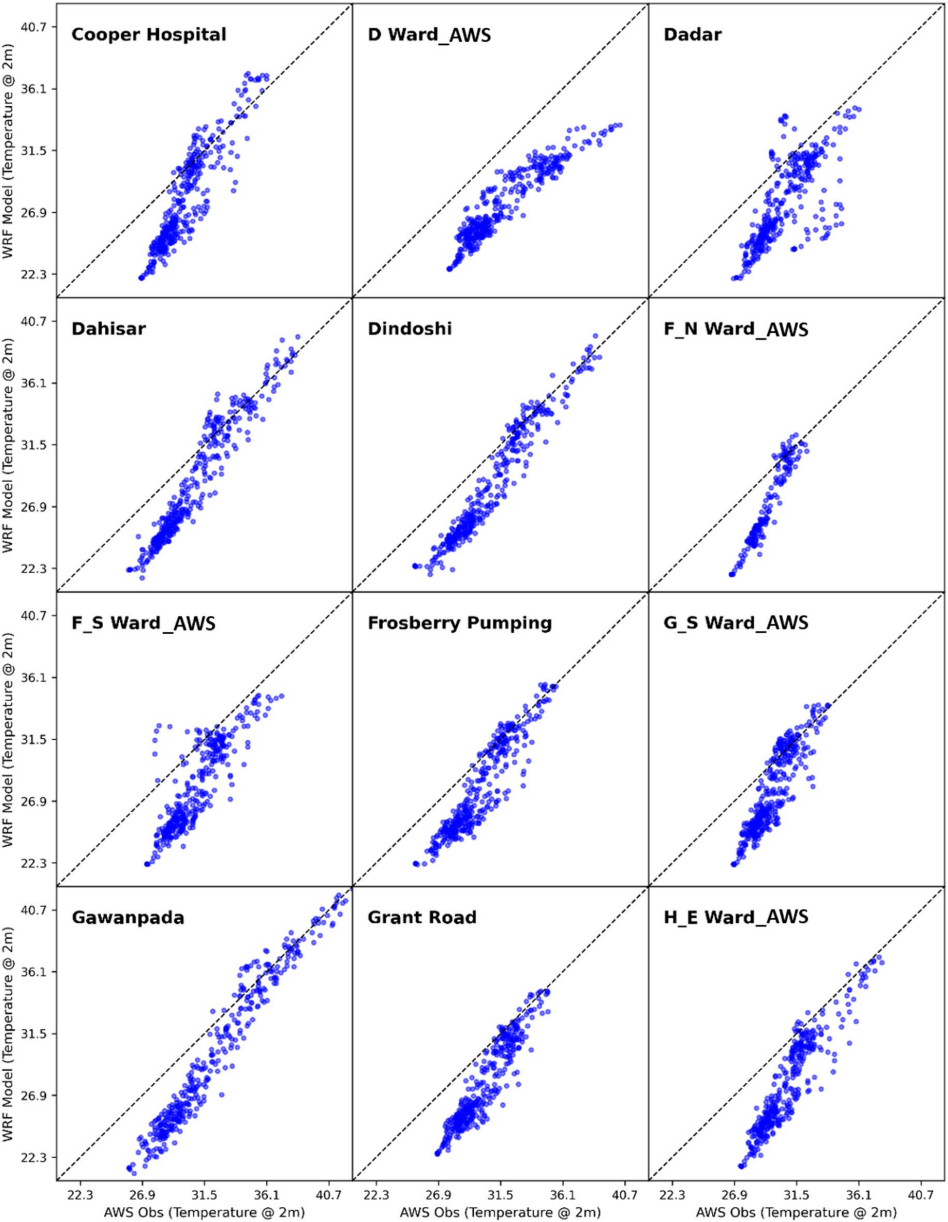
Scatter plot of WRF model simulated vs. AWS observed 2 m temperature.

**Fig. 6:**
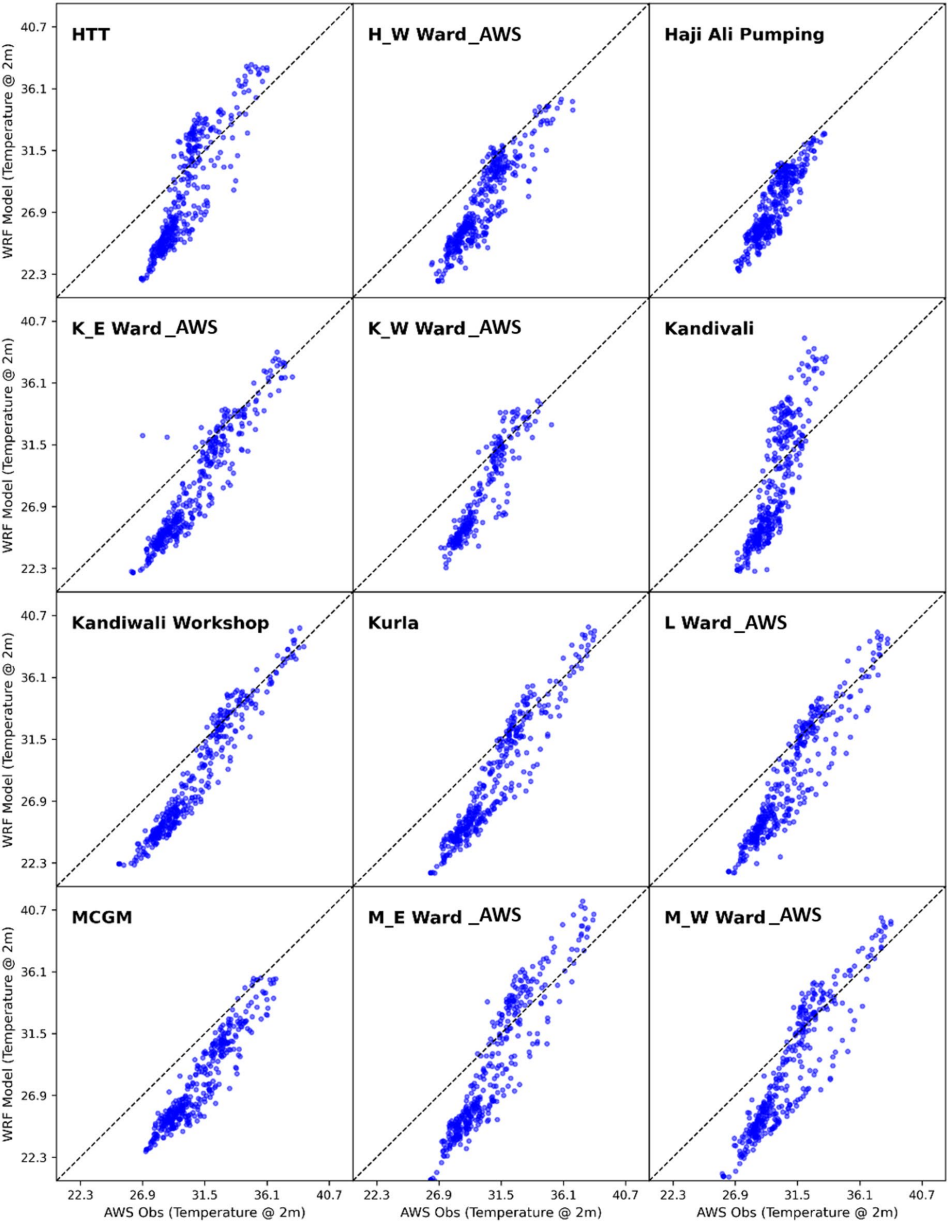
Scatter plot of WRF model simulated vs. AWS observed 2 m temperature.

To evaluate the model’s accuracy and performance, the hourly simulated values of air temperature at 2 m ($\:{\mathrm{T}}_{2}$), relative humidity ($\:RH$) and wind speed at 10 m ($\:WSP{D}_{10}$) from the current WRF-UCM experiment run were compared with the actual observations from 36 automatic weather stations which are located across different wards of Mumbai Metropolitan Region. From Figs. 4, 5 and 6, it can be observed from the scatter plots of WRF-UCM model output of temperature variable at 2 m with that of the AWS observed values of temperature, that the model is predicting accurately for the higher values but it is under predicting at the lower values which is during the night time because of the stable condition model is unable to predict the correctly. Throughout the simulation it was found the maximum UTCI values were usually achieved at 0700 UTC, hence the 0700 UTC was considered for further calculations. Statistical techniques such as RMSE, PCC, MAE and IOA (as detailed in Supplementary_Material Appendix C) were used for assessment.

### Heat hazard

**Fig. 7:**
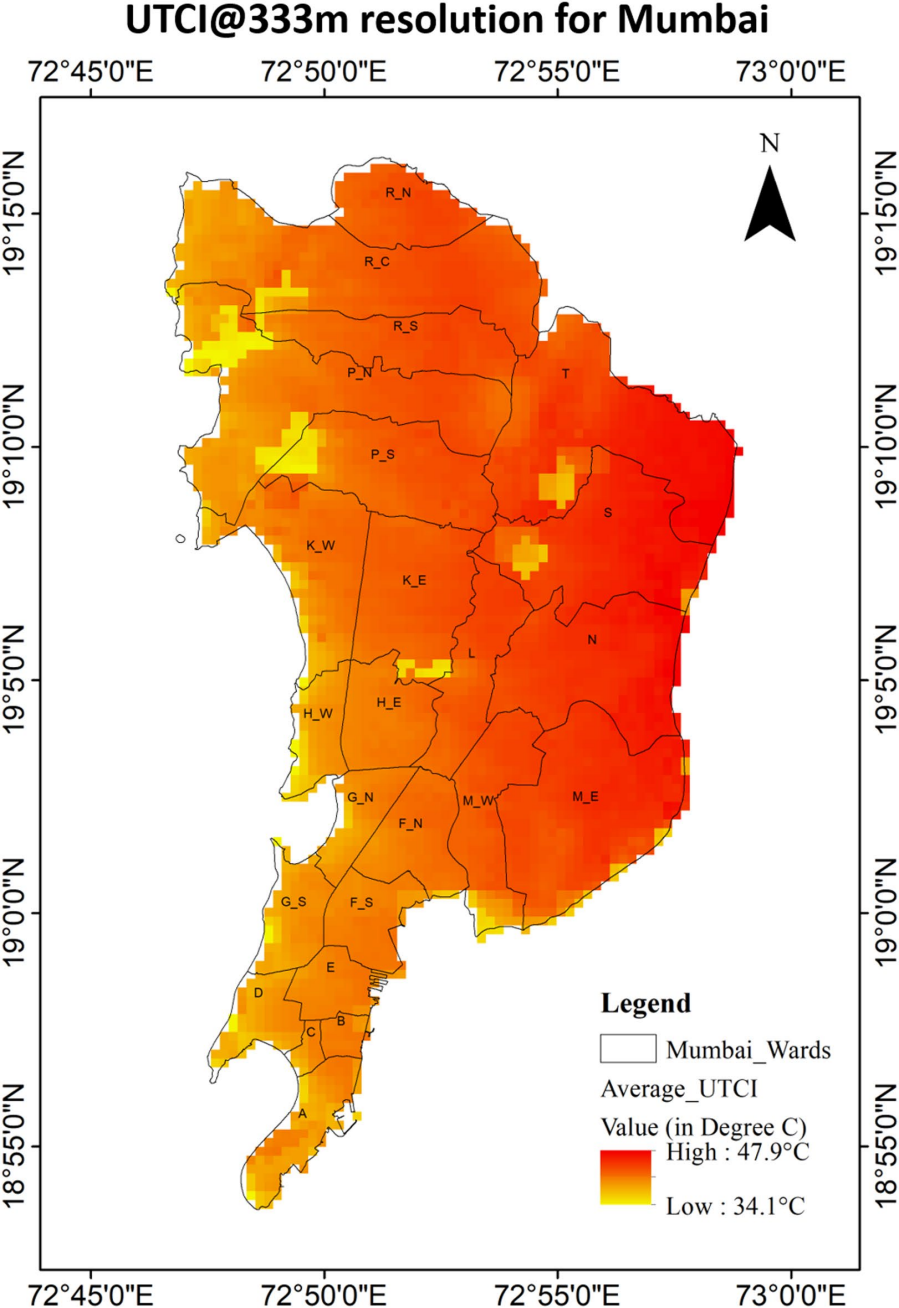
UTCI map of Mumbai at 333 m resolution.

**Fig. 8:**
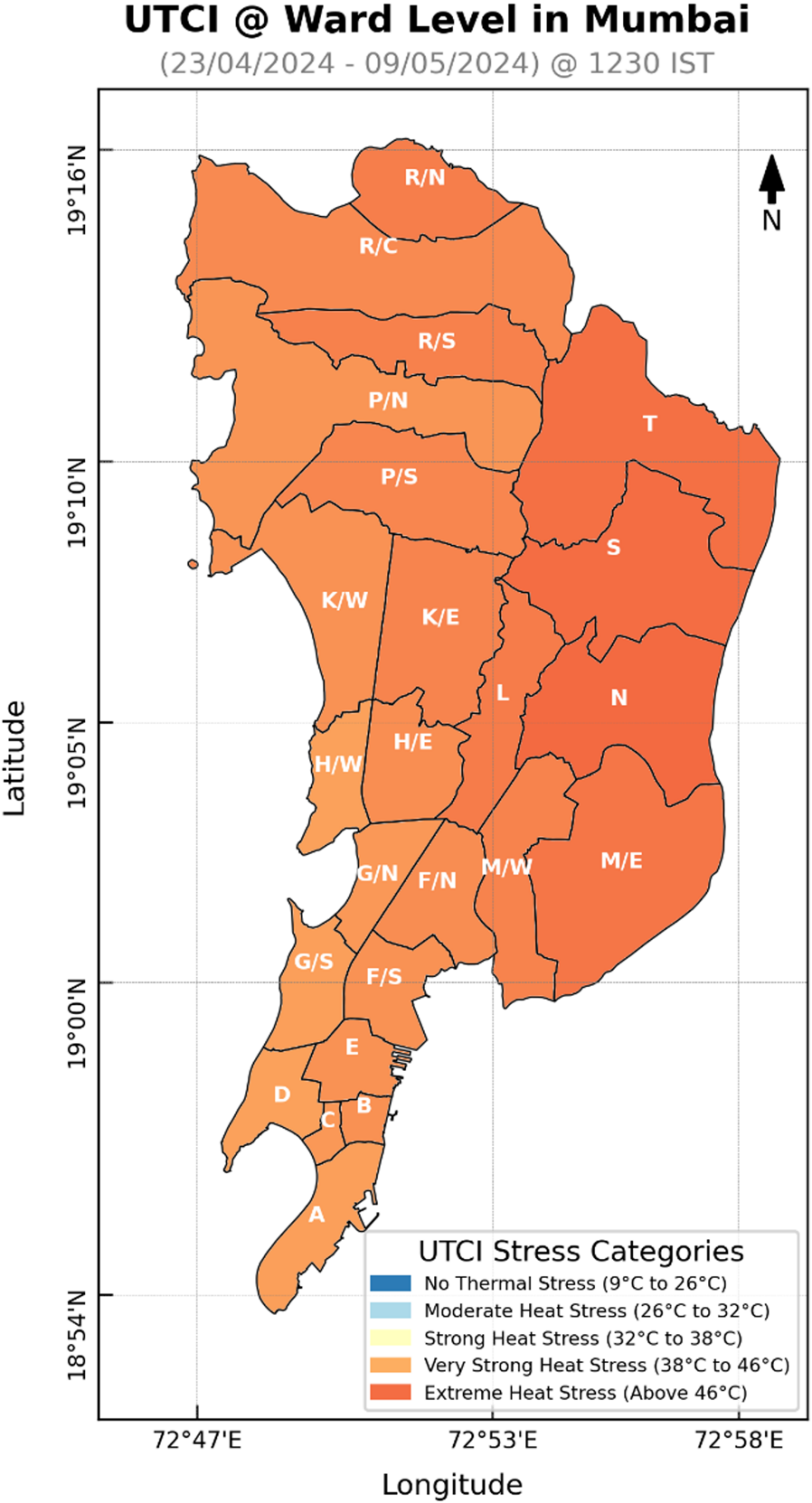
Average UTCI values at the ward level in Mumbai.

Figures 7 and 8 illustrates the heat stress conditions experienced in Mumbai between April 23 and May 9, 2024, at 0700 UTC (i.e., 1230 IST). During this fifteen-day period, the Universal Thermal Climate Index (UTCI) ranged from 34.1 °C to 47.9 °C, making a gap of 13.8 °C. As the vulnerability and exposure data are at the ward level, hence the UTCI for each ward was averaged which resulted in a range starting from 39 °C to 45.1 °C, indicating a 6.1 °C variation across the municipal boundaries of Mumbai. The city predominantly faced “Very Strong Heat Stress” posing significant health risks to residents. All areas of the Mumbai fell under the “Very Strong Heat Stress” category, with the eastern regions being notably warmer compared to the western coastal zones. The highest temperatures were observed in the easternmost wards—N, S, and T—while the coolest zones were in the south and south western coastal wards— A, D, G/S and H/W. The analysis revealed a noticeable 6.1 °C variation in UTCI across the city, with specific pockets showing extreme differences.

**Fig. 9:**
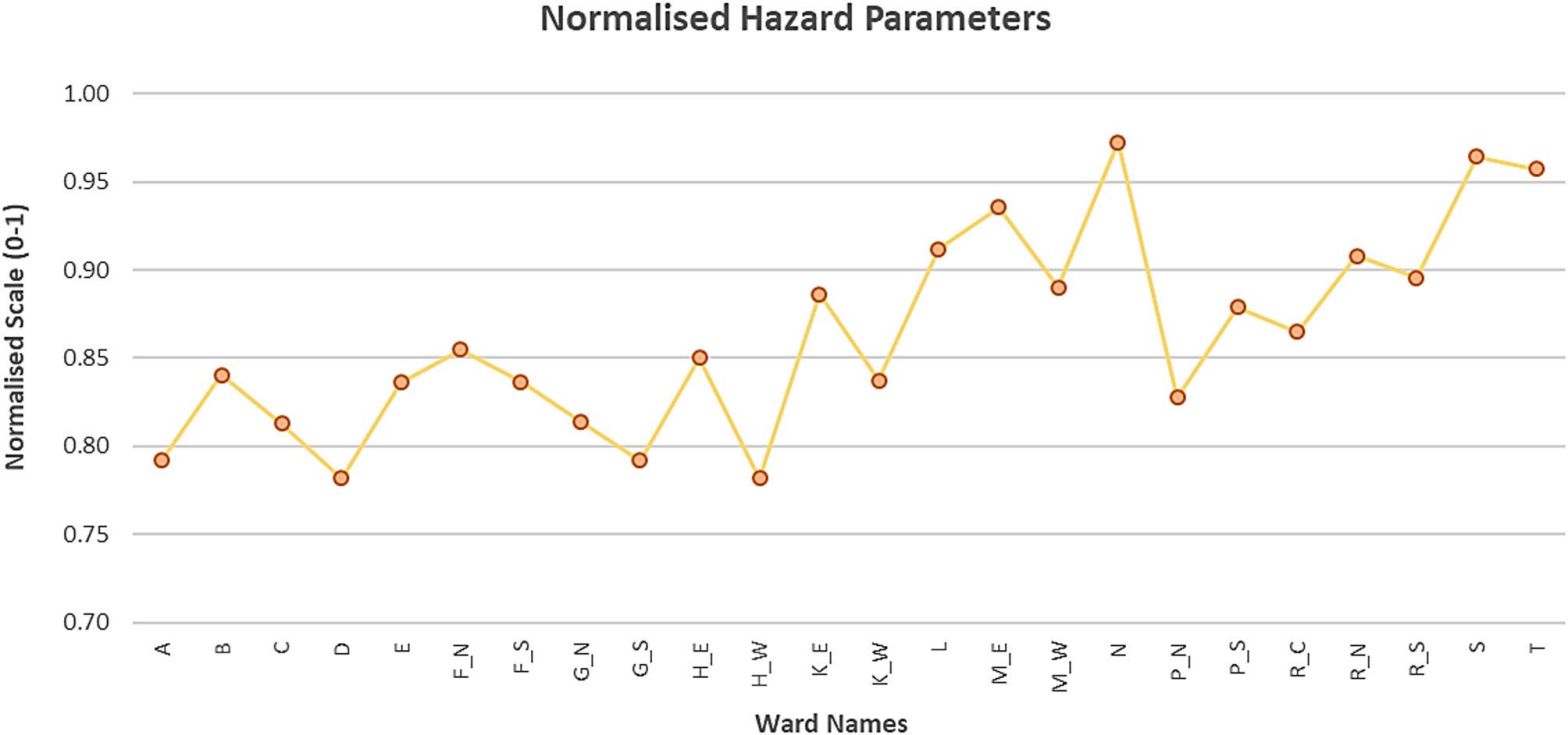
Normalized hazard parameters showing the categorically normalized UTCIs from 0 to 1 in each ward of MCGM equally distributed over x-axis.

**Fig. 10:**
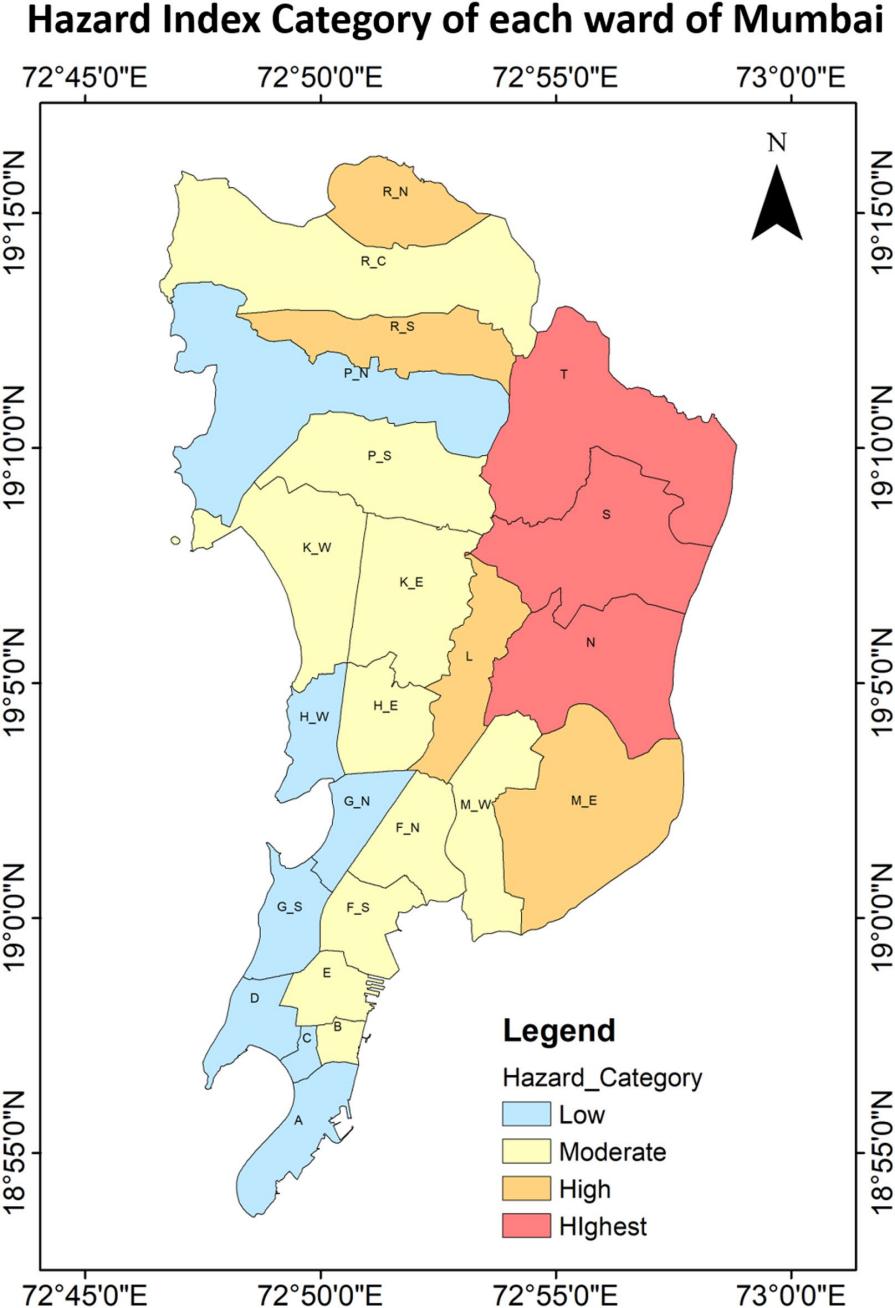
Spatial distribution of the heat stress hazard index for each ward of Mumbai.

Figure 9 represents normalized UTCI values across wards, ranging from 0.78 to 0.97. All these values are within 0.75–1.00 range, confirm that each and every ward in the city experienced “Very Strong Heat Stress” during this timeframe. Figure 10 further categorizes the city’s wards based on heat stress levels: it can be observed that 100% of the city wards experienced the high stress as per the Hazard category classification. The eastern wards being notably experienced high UTCI heat stress compared to the western coastal wards in Mumbai, but still lies in the “very strong heat stress” category. The eastern parts continued to show relatively higher thermal stress.

Once these ward values were classified, it was seen that 12.5% or 3 number of the wards lie in the “Highest” category (ward N, S and T). 16.7% or 4 wards lie in the “High” category of Hazard Index. 41.7% or 10 wards lied in the “Moderate” category, while 29.1% of wards lie in the “Low” category. There was no “Lowest” category in the graph. Through Figs. 7, 8 and 10, it was also observed that the wards near the coast faced lesser heat stress hazard than the wards farther from the sea. The Eastern region houses all the “Highest” hazard zones, while the Western region house all the “Low” hazard zones.

### LCZ, LST and UTCI comparison

**Fig. 11:**
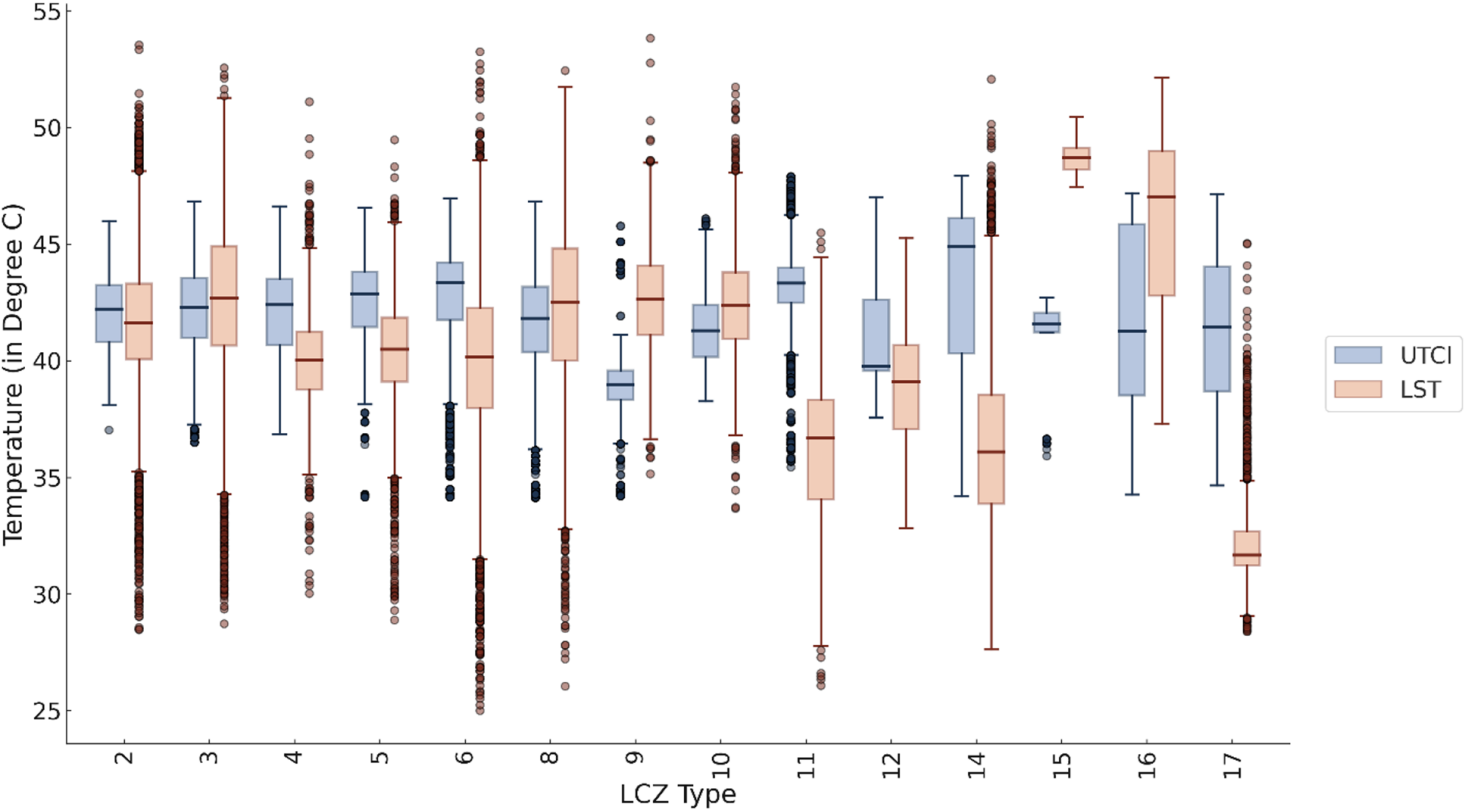
Comparative analysis of LST and UTCI data for each LCZ type in Mumbai.

By comparing the LST and UTCI data (Fig. 11), it was observed that in the built areas with high compactness (LCZ2 and LCZ3) the LST and UTCI have nearly same means (42.05 °C and 41.62 °C, UTCI and LST respectively for LCZ2 and 42.27 °C and 42.75 °C, UTCI and LST respectively for LCZ3), as the compactness factor decreases (LCZ 4, LCZ 5 and LCZ 6), the gap between UTCI and LST increases than that in comparison to compact LCZ, in these cases the absolute value of UTCI is nearly same of higher than the LST. Mean UTCI for LCZ 4, LCZ 5 and LCZ 6 reached to a value of 42.24 °C, 42.80 °C and 42.75 °C respectively, while for LST the means in LCZ 4 reached at 40.08 °C, for LCZ 5, it went to 40.37 °C and for LST 6, it became 40.03 °C. LCZ 8 and LCZ 10, large low-rise structure and heavy industries respectively, show the same means as compact areas showed with mean LST reaching to 42.31 °C and 42.41 °C respectively and mean UTCI reaching to 41.67 °C and 41.46 °C respectively, contrary to the sparsely built structures were the LST showed high values (mean LST 42.54 °C and mean UTCI 38.76 °C).

In terms of non-built LCZ types, LCZ 11, LCZ 12 and LCZ 14, due to their green cover had a low LST values in comparison to other LCZs and also in comparison to UTCI. The mean UTCI and mean LST for LCZ 11 is 43.32 °C and 36.45 °C, while for LCZ 12 it is 40.89 °C and 38.85 °C; and in case of LCZ 14, the means are 43.13 °C and 36.51 °C respectively. While where the surfaces were bare in nature like LCZ 15 and LCZ 16, the heat phenomena resulted in higher LST values than the UTCI. For LCZ 15 the mean UTCI is 40.70 °C, while the mean LST is 48.69 °C; and for LCZ 16 the mean UTCI is 41.27 °C, while the mean LST is 45.98 °C. In case of LCZ 17 – water, it showed the similar behaviour as that of the green surfaces, where the mean LST (32.35 °C) is much lower than the other LCZ values and then the mean UTCI (40.99 °C). For this study UTCI has been taken as it is an index that shows the thermal comfort and heat perception of the human beings.

### Vulnerability index

**Fig. 12:**
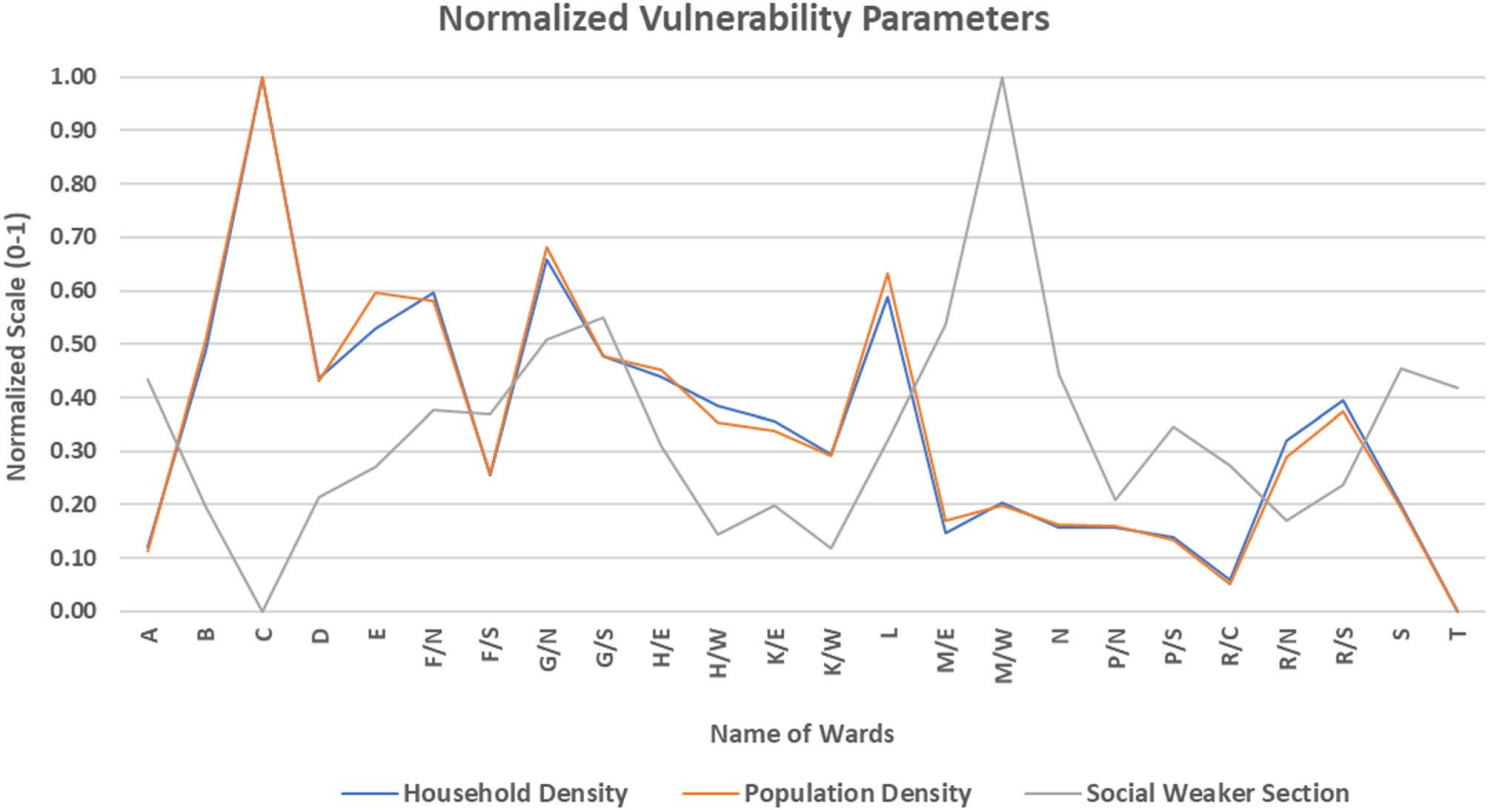
Normalized vulnerability parameters showing the normalized values for parameters from 0 to 1 in each ward of MCGM equally distributed over x-axis.

**Fig. 13:**
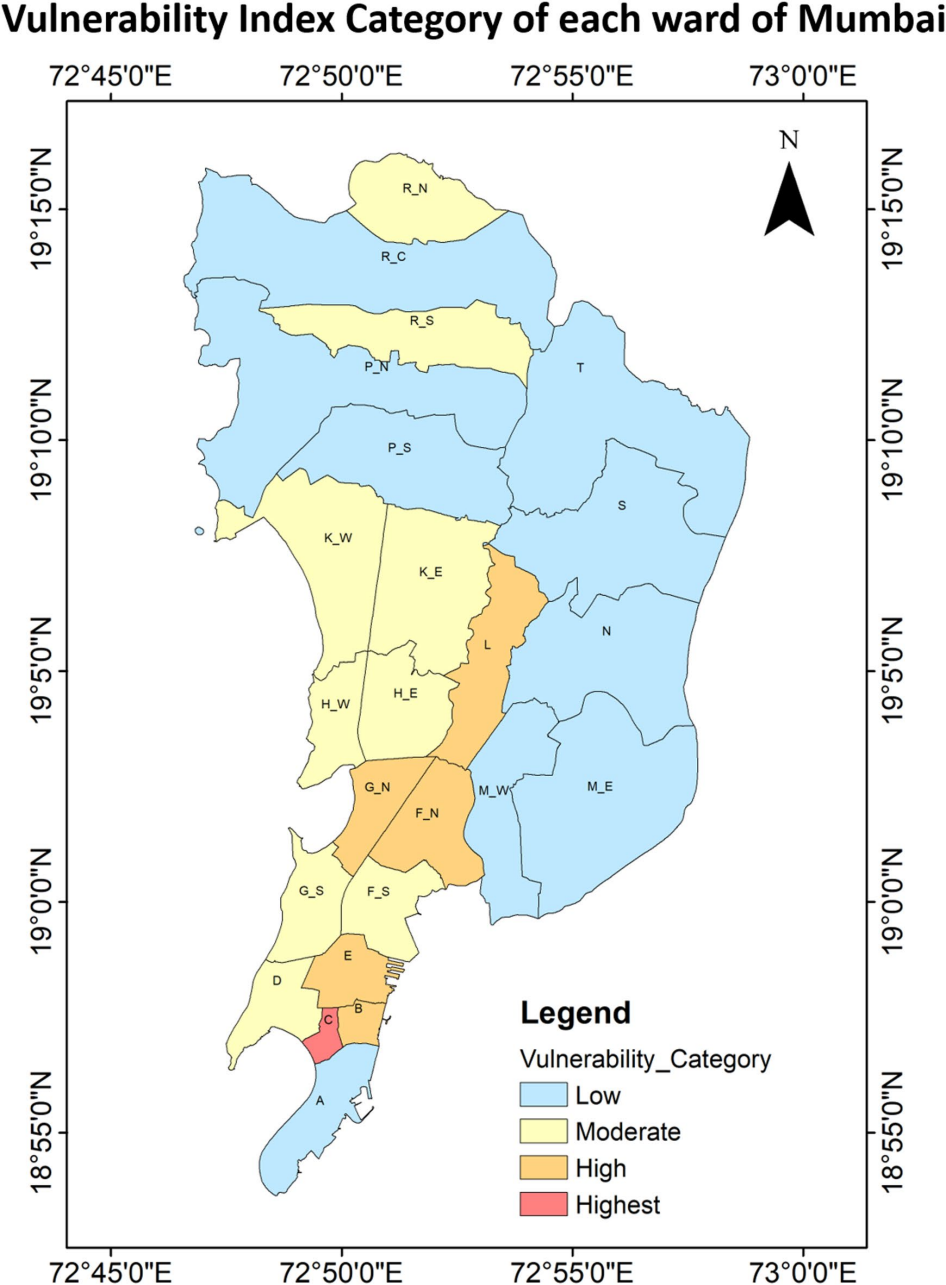
Spatial distribution of the vulnerability index category for each ward of Mumbai from the highest (5) to lowest (1).

The vulnerability conditions assessed through various parameters are depicted in Fig. 12, while Fig. 13; Table 4 outline the spatial distribution of vulnerability across all wards in Mumbai. Principal Component Analysis (PCA) was applied to three variables contributing to the vulnerability index, with results summarized in Table 5. The Kaiser-Meyer-Olkin (KMO) test yielded a value of 0.521, and Bartlett’s test of sphericity showed statistical significance with a p-value of 0.000 confirming that the variables were sufficiently correlated for PCA. PCA identified all three variables as 1 principal component (PC) that accounted for the explanation of 72.8% of the data. In this case as there was only 1 Principal Component (PC), which was formulated by taking into consideration the variability of the indicators of vulnerability. Hence, no varimax rotation could have been applied, and no component weights were used in the calculation of the vulnerability index. It was mathematically formulated using IBM SPSS Statistics 26 (IBM Corp., 2019) tool. Hence, it was directly derived from the aggregate of the standardized values of three variables. The new formulated PC was further normalised so the value ranges from 0 to 1. This normalised PC depict the Vulnerability Index. It was then classified into five categories—ranging from “Highest” to “Lowest.” According to Fig. 13 Vulnerability Category, only 4.2% of the wards i.e. ward C; is under “Highest” category. Notably, ward C had a “Lowest” vulnerability score in terms of the socially weaker section, yet the extreme densities of population and households pushed it into the highest vulnerability bracket. 20.8% of Mumbai’s wards (ward B, E, F/N, G/N and L) fall under the “High” vulnerability category. These wards showed “High” levels of population and household density. 37.5% or 9 wards were classified under the “Moderate” vulnerability category, scattered evenly across the city covering majority of the Mumbai area. While most parameters in this group scored moderate values, 1 ward (Ward G/S) had high population density, household density and social weaker section. In this ward, all three section were marked “High” but its overall impact was insufficient to elevate the ward’s vulnerability classification. Around 37.5% or 9 wards of the Mumbai city falls under the “Low” vulnerability category, while none of the wards were classified under the “Lowest” category. Among the “Low” category wards, one of them showed “Lowest” population and household density (Ward T), while 3 wards (N, S and M/E) despite having “High” category of social weaker section, were in “Low” category. Majorly the combination of “Moderate” and “Low” scores across the parameters contributed to these classifications. This highlights a challenge for heat stress mitigation and risk management, indicating a city-wide vulnerability that remains significantly high.

**Table 4:** Category distributions of heat vulnerability parameters and vulnerability indices in Mumbai wards from highest (5) to lowest (1).

Ward	A	B	C	D	E	F/*N*	F/S	G/*N*	G/S	H/E	H/W	K/E
Household density	2	4	5	3	4	4	3	4	4	3	3	3
Population density	2	4	5	3	4	4	3	4	4	3	3	3
Socially weaker section	3	2	1	2	3	3	3	4	4	3	2	2
Vulnerability index	2	4	5	3	4	4	3	4	3	3	3	3
Ward	K/W	L	M/E	M/W	N	P/N	P/S	R/C	R/N	R/S	S	T
Household density	3	4	2	2	2	2	2	2	3	3	2	1
Population density	3	4	2	2	2	2	2	2	3	3	2	1
Socially weaker section	2	3	4	5	4	2	3	3	2	2	4	3
Vulnerability index	3	4	2	2	2	2	2	2	3	3	2	2

**Table 5:** Principal component analysis (PCA) test results for the vulnerability parameters.

a. KMO and Bartlett’s test									
Kaiser-Meyer-Olkin measure of sampling adequacy									0.521
Bartlett’s test of sphericity	Approx. Chi-square								104.649
	df								3
	Sig								< 0.001
b. Component matrix									
Indicator								Component	
									1
Household density									0.973
Population density									0.968
Social weaker section									-0.548
c. Total variance explained									
Component	Initial eigen values			Extraction sums of squared loadings			Rotation sums of squared loadings		
	Total	% of variance	Cumulative %	Total	% of variance	Cumulative %	Total	% of variance	Cumulative %
1	2.184	72.814	72.814	2.184	72.814	72.814	-	-	-

### Exposure

**Fig. 14:**
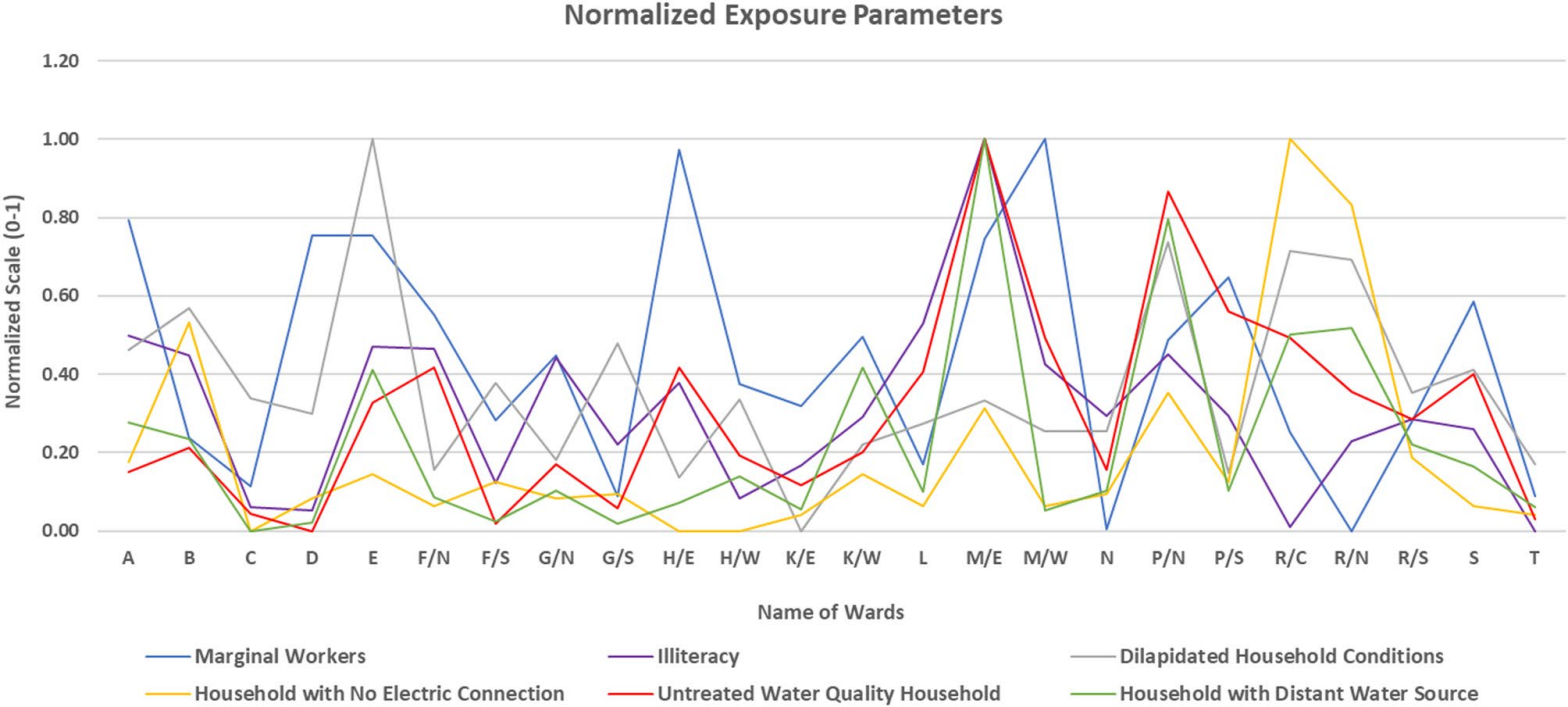
Normalized exposure parameters showing the normalized values for the parameters from 0 to 1 in each ward of MCGM equally distributed over x-axis.

**Fig. 15:**
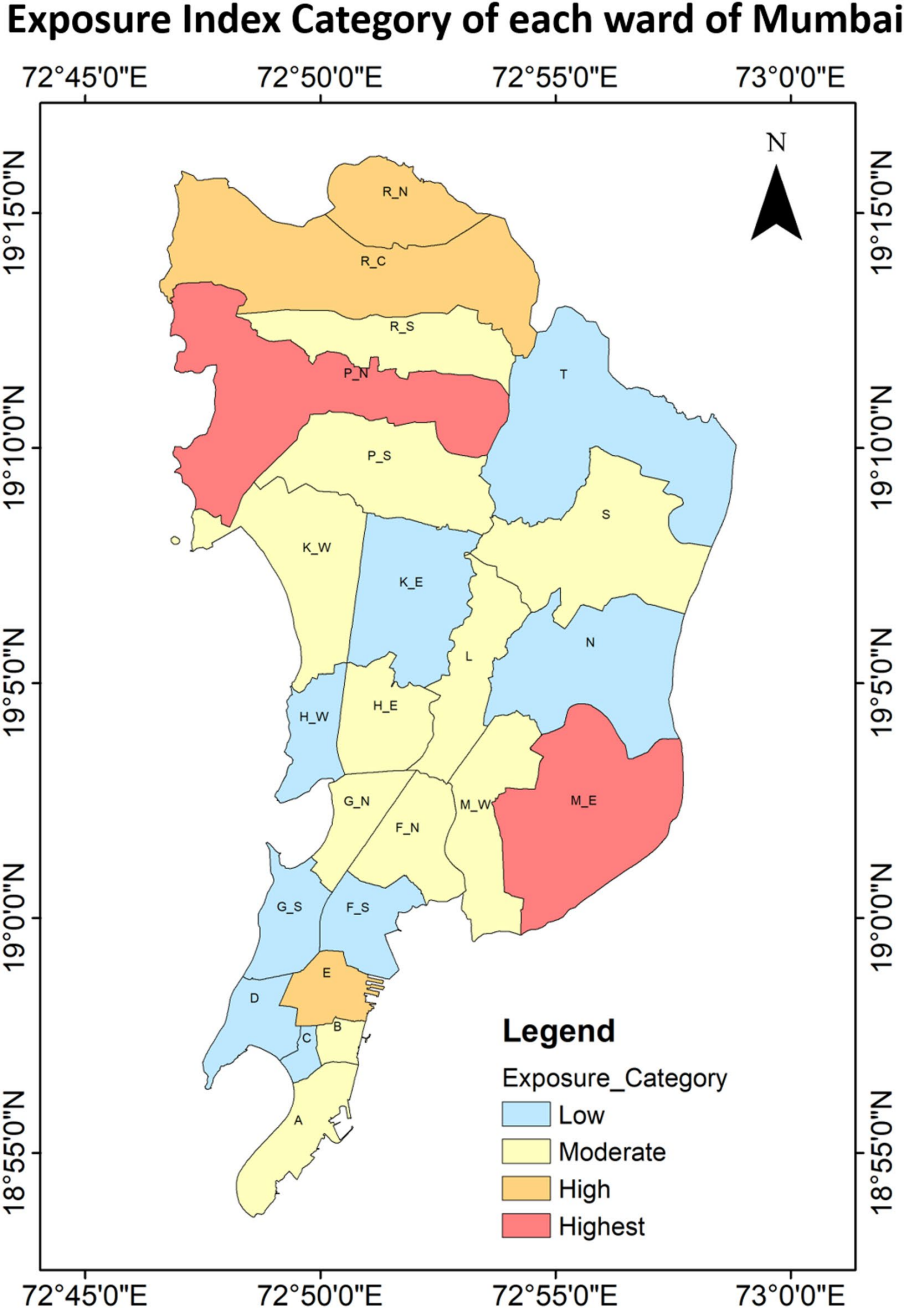
Spatial distribution of the exposure indices for each ward of Mumbai from the highest (5) to lowest (1).

Exposure levels across Mumbai’s wards were analysed using six parameters, as shown in Fig. 14; Table 6. A Principal Component Analysis (PCA) was applied to these parameters, with results detailed in Table 7. The analysis yielded a KMO value of 0.677, indicating an acceptable level of sampling adequacy. Bartlett’s test of sphericity returned a statistically significant p-value of 0.000, confirming the data’s suitability for PCA. Two principal components were identified, together explaining 76.451% of the total variance. The varimax-rotated eigenvalues were 2.326 and 2.261, confirming both components as significant. Component weights, calculated using the variance ratios, were 0.507 for Component 1 and 0.493 for Component 2. These weights were used to compute the Exposure Index using Eq. 3, which was then classified into five categories: Highest, High, Moderate, Low, and Lowest. Figure 15 highlights that 2 of the Mumbai’s wards fall into the “Highest” exposure category (Ward M/E and P/N), making it 8.3% of the wards in Mumbai. The “High” exposure category comprised 12.5% of the wards, or 3 wards, distributed across northern to southern parts of Mumbai. In this group, all the five wards have 3–4 parameters in the “Highest” or “High” category. For example, ward R/C and R/N had illiteracy rate and marginal workers in the “Low” or “Moderate” category respectively, but other parameters collectively contributed to a high exposure score. Ward P/N and M/E have many parameters like distance from water source, untreated water quality, illiteracy or dilapidated household in “Highest” category. A total of 45.8% or 11 wards were classified under the “Moderate” exposure category covering the majority of the Mumbai area. These wards typically presented a mix of extremes, with a few parameters in the “Moderate”, “High” or “Highest” range, while the remaining fell in the “Low” category. The “Low” exposure category comprises of 33.4% or 8 wards. These wards were located along the city’s periphery, particularly in the northeast part central part and in the southern part along the coastal area. In this category, ward C, K/E, and T had minimum 5 parameters in the “Low” or “Lowest” category, signifying that even “Low” exposure wards were not entirely free from significant issues. None of the wards was categorized under the “Lowest” exposure category.

**Table 6:** Category distributions of heat exposure parameters and exposure indices for Mumbai wards from the highest (5) to lowest (1).

Ward	A	B	C	D	E	F/*N*	F/S	G/*N*	G/S	H/E	H/W	K/E
Marginal workers	4	2	2	4	4	3	2	3	2	5	3	3
Illiteracy rate	4	4	2	2	4	4	2	4	3	3	2	2
Dilapidated household	3	4	3	3	5	2	3	2	3	2	3	1
No electric connection	3	4	2	3	3	2	3	3	3	2	2	2
Untreated water quality	2	3	2	2	3	3	2	2	2	3	3	2
Distant water source	3	3	2	2	4	2	2	3	2	2	3	2
Exposure index	3	3	2	2	4	3	2	3	2	3	2	2
Ward	K/W	L	M/E	M/W	N	P/N	P/S	R/C	R/N	R/S	S	T
Marginal workers	3	2	4	5	2	3	4	2	2	2	4	2
Illiteracy rate	3	4	5	4	3	4	3	2	3	3	3	2
Dilapidated household	2	3	3	2	2	5	2	5	4	3	3	2
No electric connection	3	2	3	2	3	4	3	5	5	3	2	2
Untreated water quality	3	3	5	4	2	5	4	4	3	3	3	2
Distant water source	4	2	5	2	3	5	3	4	4	3	3	2
Exposure index	3	3	5	3	2	5	3	4	4	3	3	2

**Table 7:** Principal component analysis (PCA) test results for the exposure parameters.

a. KMO and Bartlett’s test									
Kaiser-Meyer-Olkin measure of sampling adequacy									0.677
Bartlett’s test of sphericity	Approx. Chi-square								62.718
	df								15
	Sig								< 0.001
b. Component matrix									
Indicator								Component	
								1	2
Marginal work								0.728	-0.333
Illiteracy								0.873	0.065
Dilapidated household condition								0.021	0.810
Untreated water quality Household								-0.054	0.905
Household with distant water source								0.825	0.384
Household no electrical connection								0.592	0.723
c. Total variance explained									
Component	Initial eigen values			Extraction sums of squared loadings			Rotation sums of squared loadings		
	Total	% of variance	Cumulative %	Total	% of variance	Cumulative %	Total	% of variance	Cumulative %
1	2.822	47.040	47.040	2.822	47.040	47.040	2.326	38.760	38.760
2	1.765	29.411	76.451	1.765	29.411	76.451	2.261	37.691	76.451

### Heat stress risk index

**Fig. 16:**
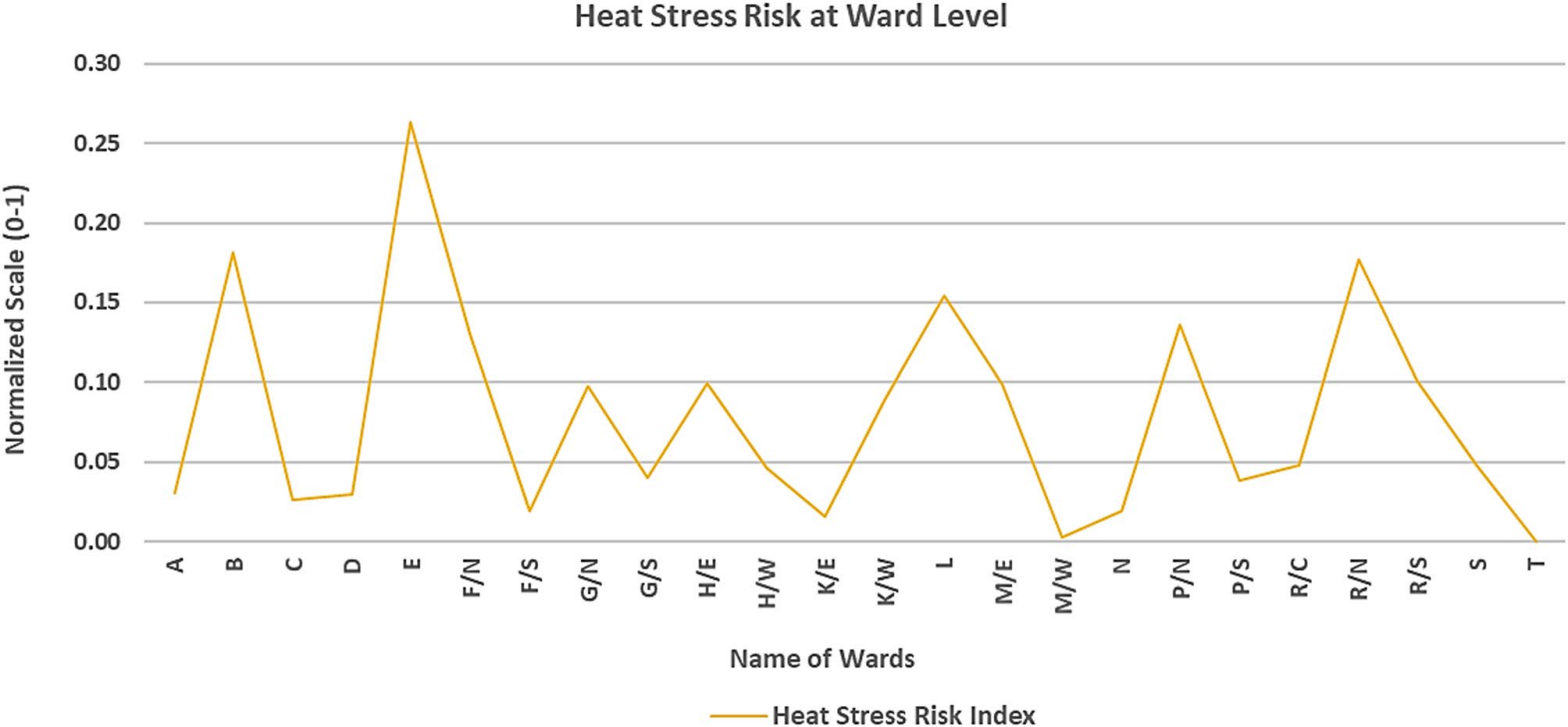
Heat stress risk indices in each ward of Mumbai equally distributed over x-axis.

**Fig. 17:**
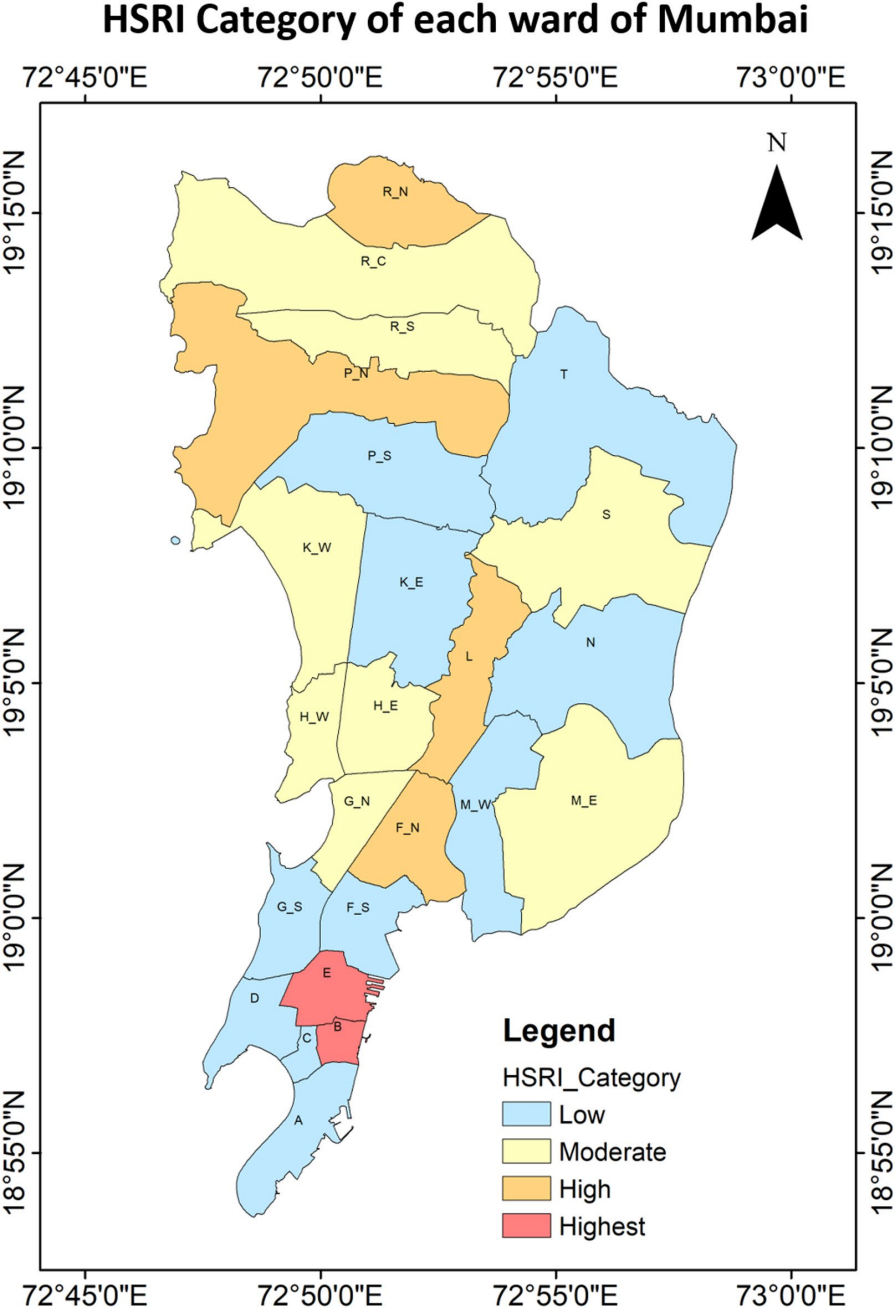
Spatial distribution of the heat stress risk index for each ward of Mumbai from the highest (5) to lowest (1).

Figures 16 and 17 present the spatial distribution of the Heat Stress Risk Index (HSRI) across Mumbai’s wards. None of the Mumbai ward were categorized as having the “Highest” HSRI. The “Highest” category encompasses 8.3% of the total number of wards making it 2 wards in number (ward B and E), while “High” HSRI category covered 16.7% of the wards, or 4 in total (ward F/N, L, P/N and R/N). Among these, all the “Highest” category ward have a “High” vulnerability category, “High” or “Moderate” exposure category and a “Moderate” hazard category and the four “High” HSRI category wards have “High” hazard index in 2 wards, “Moderate” hazard category in 1 and even “Low” hazard category in 1; two of the wards have “High” vulnerability and one of the wards have “Moderate” vulnerability, while one of the “Low” vulnerability level but had “Highest” exposure levels that raised their overall risk. Around 33.3% or 8 wards of the Mumbai covering major part of the area lies in the “Moderate” HSRI category. Meanwhile, the “Low” HSRI category included around 41.7% of the area or 10 wards, making it the highest share holder. In these “Low” risk wards, despite hazard indices was “Highest” the vulnerability and exposure indices remained in the “Low” categories, mitigating the overall risk (Ward T). Notably, none of the wards fell under the “Lowest” category. This overall implies that the Hazard index like UTCI or heat stress metric may not be fully useful to plan adaptation and mitigation policies.

## Discussion

Human health and productivity can be affected by heat stress even under conditions that are not classified as extreme. Often, heat-related mortality is underestimated and typically only recorded during heatwave events, leading to signal detection bias in health data^72,73^. Although adaptive responses and physiological acclimatization may reduce vulnerability over time^74^, if the pace of climate change exceeds the population’s ability to acclimatize, the risk of fatal outcomes increases. Additionally, acclimatization alone is not sufficient protection when individuals engage in physically demanding or complex tasks, which can lead to hyperthermia^75^.

This study identifies heat stress risk at ward scale during a year marked by record-high average global land and ocean surface temperatures^76^. While global assessments of heat stress commonly based on UTCI derived from reanalysis datasets and aggregated over broad zones—are useful for understanding large-scale patterns, while risk mapping is essential for informing local policy and urban heat management strategies. The UTCI-based heat maps Fig. 10 revealed that eastern parts of the city exhibit higher heat stress compared to the northern, western and southern region. This spatial variation is closely linked to underlying land cover: vegetation is relatively higher in the eastern and northern zones, while the western and southern parts are dominated by high built-up. Despite having high built-up area in the western and southern parts the heat stress is less that the eastern part may be due to the coastal influence. Interestingly, areas with the highest heat hazard do not necessarily coincide with the highest risk (see Figs. 10 and 16), largely due to differences in vulnerability and exposure profiles. For example, some “High” or “Highest” hazard wards may experience only moderate vulnerability and low exposure, resulting in a lower overall risk category. For areas, be classified into “Moderate” or “High” risk zones their higher vulnerability or exposure levels are to be considered along with their “Moderate” or “High” heat hazard, as illustrated in Fig. 17. This reinforces the necessity of evaluating heat risk as a composite index, integrating hazard, vulnerability, and exposure dimensions.

To address urban heat stress effectively, all wards are identified as “Highest” or “High” hazard areas which should be prioritized for nature-based or engineered solutions that reduce ambient heat levels. Similarly, wards falling into the “High” risk categories (Fig. 13) require mitigation-focused planning to address socio-economic vulnerability. Given that UTCI captures a combination of temperature, humidity, wind speed, and radiation, interventions should target one or more of these variables. For instance, increasing shaded areas can reduce solar exposure, while misting systems or dehumidifiers can help regulate humidity. Urban design strategies like optimizing street canyons may also improve airflow and reduce pollutant concentrations, thus lowering thermal stress. As this study was a prototype for the extreme event, hence a detailed analysis over the long period can be performed to understand the temporal, spatial and seasonal variations over the time to identify the locations for interventions that will help in adaptation, and mitigation of UTCI-based heat hazard, leading to increase of heat resilience in the city. In terms of social vulnerability, 29.2% and 25.0% of wards fall under the “Highest” and “High” categories for population density and concentration of socioeconomically weaker sections, respectively (Table 4). Policymakers should prioritize these wards in their action plans. Strategies may include promoting de-densification through managed migration or family planning initiatives, and fostering economic development and education in socially vulnerable communities. The physical dimensions of vulnerability represented by household density and population density further highlight these risks. Over 29.2% of wards fall into the “Highest” and “High” category for household density and population density. These areas should be targeted for improving housing quality, promoting vertical expansion, and integrating green infrastructure.

Figure 15 further highlights the spatial distribution of heat exposure indicators across the city and identifies areas where targeted interventions could significantly reduce overall exposure-related risk. Key infrastructure and service deficits such as poor housing conditions, lack of electricity, and inadequate access to treated water amplifies the impacts of extreme heat on vulnerable populations. Social aspects of exposure, particularly the proportion of marginal workers (8.3%) and illiterate residents (4.2%) across the wards, account for a “Highest” exposure category. These segments are particularly challenging to address due to the scale of the population involved. However, long-term strategies rooted in structural policy shifts, such as implementation of the National Education Policy (NEP), can progressively reduce illiteracy and improve livelihoods—thereby reducing social vulnerability to heat stress. Immediate, action-oriented solutions should prioritize the deployment of shaded public areas and community-level water access points in high-exposure zones. These simple, cost-effective interventions can drastically improve comfort and reduce the likelihood of heat-related illnesses. Several infrastructural and service-based exposure indicators stand out like Dilapidated household conditions in 12.5% of wards, Untreated water supply households in 8.3%, Households with distant water sources in 8.3% and Households with no electricity connections in 8.3% of wards which account for a “Highest” exposure category. Addressing these deficiencies requires collaborative efforts among urban planners, architects, policymakers, and community-based organizations. For example, initiatives like the Light House Project aim to improve household infrastructure through rapid, affordable housing solutions. Similarly, improved water access can be ensured via large-scale rainwater harvesting systems or decentralized water storage facilities. Promotion of solar power through India’s national solar mission can also ensure sustainable and independent energy access in high-risk areas. City development policies must prioritize intervention based on all four index categories (hazard, vulnerability, exposure, and risk).

The UTCI hazard map generated using the WRF-UCM model (Figs. 7 and 8) serves as a key output of this study. With a spatial resolution of 333 m, it integrates meteorological variables, urban morphology, and Local Climate Zone (LCZ) data to provide a nuanced understanding of urban heat distribution. The study confirms that Mumbai, as a highly urbanized region, remains extensively impacted by heat stress during heat stress event of 2024.

World Urban Database and Access Portal Tools (WUDAPT) utilises satellite imagery and machine learning techniques (random forest) to calculate the LCZ. This is globally a well-accepted technique, but various authors are improving the technique even further to get more accurate results. Wellinger, et al., (2024) combined geodata of urban canopy parameters with the remote sensing-based LCZ map of Bern, Switzerland^77^. This helped in more accurate description of water surfaces, non-built areas, and building height. This could be helpful for cities having available urban canopy parameter datasets. Lefevre, et al., (2025) utilised 159 training areas by using Google Earth, in-situ measurements, and field knowledge to classify these areas into distinct LCZ categories^78^. This include average building height and building surface fraction using the GIS data from French National Institute of Geographic Information (IGN) open-access database. Kotharkar, et al., (2020) utilised the Technique for Order Preference by Similarity to the Ideal Solution (TOPSIS) method to rank the importance of each LCZ^79^. The researchers also developed new LCZ types by combining 2 types of LCZ to understand the city morphology. These techniques can be utilised in future studies to make the results more accurate. Policy recommendations derived from this analysis includes the expanding and strategically locating urban green spaces to modify local LCZs. The study discusses the impact of heat stress event in April-May 2024, with longitudinal analysis in future covering multi-seasonal and temporal analysis over the study region to understand the typical hazard conditions for locating generalised areas of heat stress hotspots to develop strategies that help in to plan, adapt and mitigate the heat stress hazard. Enhancing healthcare infrastructure in underserved wards, addressing social inequities by uplifting marginalized and illiterate populations, upgrading dilapidated housing and promoting vertical, climate-adaptive developments, ensuring access to treated water, reducing dependency on distant sources and Providing electric connections for improved living conditions and climate resilience are few of the recommendations that are essential not only for mitigating present-day risks but also for building long-term urban resilience against escalating heat stress in the face of climate change.

## Conclusion

This study advances a replicable framework for the development of the Heat Stress Risk Index (HSRI), utilizing an innovative blended approach that integrates Numerical Weather Prediction (NWP) models with census data to effectively identify urban zones with high heat stress risk. By employing the Universal Thermal Climate Index (UTCI) as a hazard index, this methodology facilitates the generation of high-resolution (333 m) Hazard maps and then to integrate the ward level hazard, vulnerability and exposure levels to generate the ward level risk map, making it a novel and useful tool for understanding heat stress risk at a ward scale. This approach addresses the critical need for a ward level heat stress risk distribution model that spans the entire urban region, confirming comprehensive identification of high-risk wards.

Using Mumbai, India, as a case study, this research contributes to advancing the application of the UTCI by integrating urban fabric elements into the model, enhancing its robustness for identifying and assessing heat stress risks in rapidly urbanizing areas. By calculating the UTCI through the WRF-UCM model and utilizing vulnerability and exposure components sourced from the Census of India 2011, this study successfully outlines a framework for understanding heat stress risks at the ward level. Principal Component Analysis (PCA) was applied to cluster and reduce correlated parameters, organizing them into principal components for both vulnerability and exposure indices. Population density, Household density and Socio-economic status parameters were used to characterise the Vulnerability factors. However, the Exposure factors are analysed by including six parameters consisting of the number of marginal workers, illiteracy rates, inadequate housing conditions, untreated water quality, distant water sources, and lack of electrical connections. The study calculated the normalized hazard, vulnerability, and exposure indices and combined them to form the HSRI, which was categorized into five segments: “Lowest” (0% of total wards), “Low” (41.7% of total wards), “Moderate” (33.3% of total wards), “High” (16.7% of total wards), and “Highest” (8.3% of total wards). These categories provide clear insights into the “Highest” and “High” risk areas, which require urgent attention to safeguard citizens from heat stress risks.

A key limitation of this study is the scale of the analysis. While the UTCI was calculated at a 333 m resolution, many of the vulnerability and exposure parameters were available only at the ward level. This restricts the resolution of the analysis and limits the granularity of the results. Future research could enhance this framework by including additional parameters, such as employment, income, community participation, and access to air conditioning, as well as by integrating exposure indices like age distributions and mortality rates. Other parameters like elderly population, health infrastructure, income, etc. are equally important, but due to non-availability of the data the vulnerability parameters got restricted to chosen 3 values. With the release of new census data, the same methodology can be deployed to provide up-to-date results. The future studies can be planned to assess the UTCI over a longer temporal and seasonal range to more accurately identify the areas that require interventions to reduce the hazard values. Despite these limitations, the results from this study are acceptable and can serve as an additional tool for urban heat stress risk management. This approach offers significant potential for stakeholders, including planners, architects, policymakers, and urban designers, to develop sustainable, heat-resilient, and disaster-resistant city planning strategies. By identifying areas most vulnerable to heat stress, this framework can help guide development policies aimed at mitigating heat-related risks and enhancing the overall resilience of urban areas against future climate extremes.

The data and code used in this study will be made available from the corresponding author upon reasonable request.

## Supplementary Information

Below is the link to the electronic supplementary material.

Supplementary Material 1

## Data Availability

The data and code used in this study will be made available from the corresponding author upon reasonable request.
