# Supplementary material for: A framework for impact based heat stress warning system for a coastal city in India
Source: Sci Rep. 2026 Mar 6;16:12254. doi: 10.1038/s41598-026-38639-9 (PMC13079777; doi:10.1038/s41598-026-38639-9)
Supplement: Supplementary file 1 — Supplementary Material 1 [file 41598_2026_38639_MOESM1_ESM.docx]

**A framework for Impact Based Heat Stress Warning System for a Coastal City in India**

**Supplementary Material**

**Appendix A**

The Universal Thermal Climate Index (UTCI) expresses thermal conditions as an equivalent temperature under reference conditions that would produce the same physiological response as the actual environment. It can differ from the actual air temperature (Ta) due to influences from factors such as wind speed ($v_{a}$), humidity (represented by vapor pressure, ($V_{p}$)), and mean radiant temperature ($T_{mrt}$) (Havenith & Fiala, 2015). The UTCI is calculated as:

$$\begin{aligned} UTCI=f\left( T_{a};T_{mrt};v_{a};V_{p} \right)=T_{a}+Offset\left( T_{a};T_{mrt};v_{a};V_{p} \right)\#\left( A1 \right) \end{aligned}$$

Representative outdoor activity:

It assumes a person is walking at a speed of 4 km/h with a metabolic heat production of 2.3 MET, equivalent to 135 W/m².

Reference Environment for UTCI:

- Wind Speed ($v_{a}$): Set at 0.5 $m/s$ at a height of 10 meters, which corresponds approximately to 0.3 $m/s$ at 1.1 meters.
- Mean Radiant Temperature ($T_{mrt}$): Assumed to be equal to the air temperature under reference conditions.
- Vapor Pressure ($V_{p}$): Corresponds to 50% relative humidity ($RH$). For higher air temperatures (above 29 $^{\circ}C$), the reference humidity is defined by a constant vapor pressure of 20 $hPa$.

The UTCI is calculated using a tool developed by (Bröde et al., 2009), which takes the following meteorological parameters as input:

- Air Temperature ($T_{a}$): Ranging from -50 $^{\circ}C$ to 50 $^{\circ}C$
- Mean Radiant Temperature Difference ($T_{mrt}-T_{a}$): From -30 $^{\circ}C$ to +70 $^{\circ}C$
- Wind Speed ($v_{a}$) at 10 meters above ground: From 0.5 to 30.3 $m/s$
- Relative Humidity ($RH$): Between 5% and 100%

Where:

- $T_{a}$ is the air temperature at 2 $m$,
- $T_{mrt}$ is the mean radiant temperature,
- $V_{a}$ is the wind speed at 10 $m$ height, and
- $RH$ is the relative humidity 2 $m$.

**Appendix B**

Most meteorological parameters required for UTCI calculation—such as air temperature ($T_{a}$), wind speed ($v_{a}$), and relative humidity ($RH$)—were directly extracted from the WRF model output using the WRF Python tool (Ladwig, 2017). However, mean radiant temperature ($T_{mrt}$), a crucial yet complex variable for assessing human thermal comfort, required additional estimation methods (Kántor & Unger, 2011). $T_{mrt}$represents the total radiation received by an object from all directions (Di Napoli et al., 2020). It can be understood as the equivalent temperature of a hypothetical black-body enclosure that emits the same net radiation as the actual environment (Kántor & Unger, 2011). In this study, a novel approach was developed to compute $MRT$at a high spatial resolution of 333 meters using outputs from the WRF-UCM model. The estimation follows the equation proposed by (Matzarakis et al., 2010):

$$\begin{aligned} \text{MRT}^{*}=\left\{ \frac{1}{\sigma}\left[ f_{a}L_{\text{surf}}^{\text{dn}}+f_{a}L_{\text{surf}}^{\text{up}}+\frac{\alpha_{\text{ir}}}{\epsilon_{p}}\left( f_{a}S_{\text{surf}}^{\text{dn,diffuse}}+f_{a}S_{\text{surf}}^{\text{up}}+f_{p}I^{*} \right) \right] \right\}^{0.25}\#\left( B1 \right) \end{aligned}$$

Where $f_{a}$ is the angle factor that is set to 0.5 for an unobstructed flat site assuming human presence and $f_{p}$ is the surface projection factor, determined by the equation:

$$\begin{aligned} f_{p}=0.308\cos\left( \gamma\left( 0.998-\frac{\gamma^{2}}{50000} \right) \right)\#\left( B2 \right) \end{aligned}$$

where γ is the solar elevation angle in degrees, calculated from the WRF model output. $L_{\text{surf}}^{\text{dn}}$ and $L_{\text{surf}}^{\text{up}}$​ are downwelling and upwelling thermal radiation components. $S_{\text{surf}}^{\text{dn,diffuse}}$represents diffuse solar radiation, while $S_{\text{surf}}^{\text{up}}$​ accounts for surface-reflected solar radiation. I* is the direct solar radiation component. $\sigma$ is the Stefan–Boltzmann constant (5.67 × 10⁻⁸ W/m²K⁴). $\alpha_{\text{ir}}$​ is the solar radiation absorption coefficient for the human body, set to 0.7. $\epsilon_{p}$​ is the emissivity of clothed human skin, with a standard value of 0.97 (Di Napoli et al., 2020).

**Appendix C**

Mathematical models used for validation:

$$\begin{aligned} PCC=\frac{\left( n\sum RS \right)-\left( \sum R\sum S \right)}{\sqrt{\left( n\sum R^{2}-\left( \sum R \right)^{2} \right)\left( n\sum S^{2}-\left( \sum S \right)^{2} \right)}}\#\left( C1 \right) \end{aligned}$$

$$\begin{aligned} MAE=\frac{\sum_{i=1}^{n} \left| S_{i}-R_{i} \right|}{n}\#\left( C2 \right) \end{aligned}$$

$$\begin{aligned} RMSE=\sqrt{\frac{\sum_{i=1}^{n} \left( S_{i}-R_{i} \right)^{2}}{n}}\#\left( C3 \right) \end{aligned}$$

$$\begin{aligned} IoA=1-\frac{\sum\left( S_{i}-R_{i} \right)^{2}}{\sum\left( \left| S_{i}-\bar{R} \right|+\left| R_{i}-\bar{R} \right| \right)^{2}}\#\left( C4 \right) \end{aligned}$$

**Temperature Data**

**Table C1:** Table shows the model performance at various stations based on RMSE, PCC, MAE, and IOA metrics.

| **Station Name** | **Andheri** | **B Ward** | **Bandhup Complex** | **Bandra** | **Britania Pumping** | **Byculla** |
| --- | --- | --- | --- | --- | --- | --- |
| **RMSE** | 2.677 | 2.876 | 2.622 | 3.280 | 2.628 | 2.830 |
| **PCC** | 0.960 | 0.626 | 0.961 | 0.887 | 0.898 | 0.913 |
| **MAE** | 2.341 | 2.539 | 2.346 | 2.818 | 2.335 | 2.449 |
| **IOA** | 0.850 | 0.642 | 0.860 | 0.666 | 0.785 | 0.759 |
| **Station Name** | **C Ward** | **CIDM** | **Chembur** | **Chincholi** | **Colaba** | **Colaba Pumping** |
| **RMSE** | 3.001 | 2.851 | 3.091 | 3.279 | 2.789 | 2.520 |
| **PCC** | 0.912 | 0.858 | 0.936 | 0.962 | 0.914 | 0.889 |
| **MAE** | 2.837 | 2.541 | 2.634 | 2.927 | 2.599 | 2.263 |
| **IOA** | 0.677 | 0.769 | 0.820 | 0.799 | 0.689 | 0.691 |
| **Station Name** | **Cooper Hospital** | **D Ward** | **Dadar** | **Dahisar** | **Dindoshi** | **F_N Ward** |
| **RMSE** | 3.030 | 4.441 | 3.732 | 2.827 | 2.790 | 2.952 |
| **PCC** | 0.911 | 0.939 | 0.734 | 0.964 | 0.971 | 0.959 |
| **MAE** | 2.583 | 4.329 | 3.277 | 2.443 | 2.416 | 2.513 |
| **IOA** | 0.744 | 0.633 | 0.615 | 0.860 | 0.866 | 0.638 |
| **Station Name** | **F_S Ward** | **Frosberry Pumping** | **G_S Ward** | **Gawanpada** | **Grant Road** | **HTT** |
| **RMSE** | 3.492 | 2.652 | 2.791 | 3.134 | 2.944 | 3.171 |
| **PCC** | 0.863 | 0.932 | 0.893 | 0.979 | 0.942 | 0.882 |
| **MAE** | 3.140 | 2.239 | 2.353 | 2.683 | 2.581 | 2.833 |
| **IOA** | 0.668 | 0.810 | 0.689 | 0.896 | 0.737 | 0.744 |
| **Station Name** | **H_E Ward** | **H_W Ward** | **Haji Ali Pumping** | **K_E Ward** | **K_W Ward** | **Kandivali** |
| **RMSE** | 3.660 | 3.084 | 2.757 | 3.142 | 2.935 | 3.461 |
| **PCC** | 0.930 | 0.913 | 0.894 | 0.946 | 0.922 | 0.865 |
| **MAE** | 3.241 | 2.690 | 2.416 | 2.738 | 2.508 | 3.140 |
| **IOA** | 0.689 | 0.719 | 0.622 | 0.807 | 0.722 | 0.631 |
| **Station Name** | **Kandiwali Workshop** | **Kurla** | **L Ward** | **MCGM** | **M_E Ward** | **M_W Ward** |
| **RMSE** | 2.750 | 3.226 | 3.288 | 3.462 | 3.279 | 3.278 |
| **PCC** | 0.970 | 0.953 | 0.935 | 0.944 | 0.936 | 0.924 |
| **MAE** | 2.366 | 2.772 | 2.788 | 3.208 | 2.917 | 2.808 |
| **IOA** | 0.873 | 0.820 | 0.800 | 0.714 | 0.824 | 0.814 |
| **Station Name** | **Malad** | **Malbar Hill** | **Malvani** | **Mandavi** | **Marol** | **Memonwada** |
| **RMSE** | 2.885 | 3.513 | 2.805 | 2.965 | 3.142 | 3.203 |
| **PCC** | 0.896 | 0.879 | 0.963 | 0.851 | 0.960 | 0.901 |
| **MAE** | 2.533 | 3.134 | 2.415 | 2.517 | 2.695 | 2.693 |
| **IOA** | 0.787 | 0.644 | 0.816 | 0.735 | 0.847 | 0.686 |
| **Station Name** | **Mulund** | **N Ward** | **Nariman** | **Rawali** | **S Ward** | **SWD** |
| **RMSE** | 1.578 | 3.551 | 3.048 | 2.979 | 2.934 | 2.891 |
| **PCC** | 0.974 | 0.902 | 0.940 | 0.873 | 0.967 | 0.859 |
| **MAE** | 1.275 | 2.980 | 2.866 | 2.547 | 2.490 | 2.466 |
| **IOA** | 0.972 | 0.798 | 0.720 | 0.796 | 0.891 | 0.689 |
| **Station Name** | **SWM Santaceuz** | **Versova Pumping** | **Vikhroli** | **Ville Parle** | **Wadala** | **Worli** |
| **RMSE** | 3.207 | 3.097 | 3.349 | 3.736 | 3.367 | 2.776 |
| **PCC** | 0.962 | 0.903 | 0.936 | 0.955 | 0.953 | 0.868 |
| **MAE** | 2.954 | 2.617 | 2.866 | 3.480 | 2.909 | 2.347 |
| **IOA** | 0.758 | 0.758 | 0.836 | 0.738 | 0.768 | 0.652 |

**Relative Humidity Data**

**Table C2:** Same as Table C1, but for Relative Humidity.

| **Station Name** | **Andheri** | **B Ward** | **Bandhup Complex** | **Bandra** | **Britania Pumping** | **Byculla** |
| --- | --- | --- | --- | --- | --- | --- |
| **RMSE** | 13.769 | 19.851 | 12.102 | 16.894 | 12.002 | 58.835 |
| **PCC** | 0.872 | 0.496 | 0.928 | 0.744 | 0.810 | -0.675 |
| **MAE** | 11.611 | 17.842 | 10.954 | 14.512 | 10.259 | 45.644 |
| **IOA** | 0.833 | 0.481 | 0.929 | 0.648 | 0.829 | 0.282 |
| **Station Name** | **C Ward** | **CIDM** | **Chembur** | **Chincholi** | **Colaba** | **Colaba Pumping** |
| **RMSE** | 11.063 | 15.145 | 16.500 | 14.948 | 13.357 | 17.369 |
| **PCC** | 0.817 | 0.764 | 0.831 | 0.897 | 0.736 | 0.730 |
| **MAE** | 9.583 | 13.616 | 14.803 | 13.106 | 11.884 | 14.652 |
| **IOA** | 0.783 | 0.752 | 0.784 | 0.827 | 0.711 | 0.578 |
| **Station Name** | **Cooper Hospital** | **D Ward** | **Dadar** | **Dahisar** | **Dindoshi** | **F_N Ward** |
| **RMSE** | 15.710 | 14.958 | 14.756 | 16.011 | 14.009 | 12.072 |
| **PCC** | 0.800 | 0.881 | 0.692 | 0.886 | 0.904 | 0.864 |
| **MAE** | 13.855 | 12.493 | 12.903 | 13.826 | 12.507 | 11.257 |
| **IOA** | 0.736 | 0.723 | 0.691 | 0.813 | 0.857 | 0.662 |
| **Station Name** | **F_S Ward** | **Frosberry Pumping** | **G_S Ward** | **Gawanpada** | **Grant Road** | **HTT** |
| **RMSE** | 16.388 | 21.264 | 16.142 | 14.793 | 14.981 | 17.684 |
| **PCC** | 0.624 | 0.559 | 0.719 | 0.927 | 0.802 | 0.758 |
| **MAE** | 14.045 | 18.568 | 14.735 | 12.796 | 13.652 | 16.061 |
| **IOA** | 0.669 | 0.432 | 0.528 | 0.888 | 0.688 | 0.701 |
| **Station Name** | **H_E Ward** | **H_W Ward** | **Haji Ali Pumping** | **K_E Ward** | **K_W Ward** | **Kandivali** |
| **RMSE** | 14.594 | 14.694 | 13.990 | 14.721 | 14.689 | 21.260 |
| **PCC** | 0.808 | 0.746 | 0.663 | 0.863 | 0.784 | 0.666 |
| **MAE** | 13.028 | 12.382 | 11.946 | 13.228 | 12.976 | 18.833 |
| **IOA** | 0.777 | 0.761 | 0.535 | 0.825 | 0.696 | 0.471 |
| **Station Name** | **Kandiwali Workshop** | **Kurla** | **L Ward** | **MCGM** | **M_E Ward** | **M_W Ward** |
| **RMSE** | 14.544 | 17.670 | 16.226 | 16.535 | 19.988 | 17.468 |
| **PCC** | 0.898 | 0.812 | 0.833 | 0.835 | 0.740 | 0.801 |
| **MAE** | 12.745 | 15.795 | 13.760 | 14.050 | 17.656 | 14.394 |
| **IOA** | 0.849 | 0.751 | 0.798 | 0.749 | 0.717 | 0.800 |
| **Station Name** | **Malad** | **Malbar Hill** | **Malvani** | **Mandavi** | **Marol** | **Memonwada** |
| **RMSE** | 15.207 | 11.159 | 15.172 | 17.139 | 16.123 | 17.358 |
| **PCC** | 0.812 | 0.797 | 0.868 | 0.699 | 0.881 | 0.714 |
| **MAE** | 13.613 | 9.541 | 12.545 | 15.738 | 13.910 | 15.912 |
| **IOA** | 0.798 | 0.770 | 0.781 | 0.656 | 0.838 | 0.605 |
| **Station Name** | **Mulund** | **N Ward** | **Nariman** | **Rawali** | **S Ward** | **SWD** |
| **RMSE** | 14.563 | 18.057 | 12.829 | 15.274 | 15.611 | 15.179 |
| **PCC** | 0.911 | 0.789 | 0.826 | 0.731 | 0.894 | 0.702 |
| **MAE** | 12.955 | 15.772 | 10.839 | 13.299 | 13.763 | 13.758 |
| **IOA** | 0.894 | 0.774 | 0.759 | 0.826 | 0.873 | 0.659 |
| **Station Name** | **SWM Santaceuz** | **Versova Pumping** | **Vikhroli** | **Ville Parle** | **Wadala** | **Worli** |
| **RMSE** | 15.074 | 15.957 | 18.949 | 13.166 | 16.755 | 78.369 |
| **PCC** | 0.876 | 0.811 | 0.826 | 0.858 | 0.839 | 0.109 |
| **MAE** | 12.891 | 12.978 | 16.944 | 11.222 | 14.703 | 75.112 |
| **IOA** | 0.778 | 0.771 | 0.778 | 0.831 | 0.768 | 0.214 |

**Wind Data**

**Table C3:** Same as Table C1, but for Wind speed at 10 meter.

| **Station Name** | **Andheri** | **B Ward** | **Bandhup Complex** | **Bandra** | **Britania Pumping** | **Byculla** |
| --- | --- | --- | --- | --- | --- | --- |
| **RMSE** | 0.658 | 3.337 | 2.204 | 2.866 | 7.740 | 3.201 |
| **PCC** | 0.904 | 0.504 | 0.321 | 0.836 | 0.830 | 0.751 |
| **MAE** | 0.549 | 2.768 | 1.712 | 2.272 | 6.374 | 2.747 |
| **IOA** | 0.914 | 0.544 | 0.585 | 0.674 | 0.536 | 0.604 |
| **Station Name** | **C Ward** | **CIDM** | **Chembur** | **Chincholi** | **Colaba** | **Colaba Pumping** |
| **RMSE** | 5.391 | 2.226 | 1.940 | 0.947 | 2.103 | 3.451 |
| **PCC** | 0.856 | 0.312 | 0.820 | 0.823 | 0.855 | 0.816 |
| **MAE** | 4.666 | 1.580 | 1.656 | 0.752 | 1.665 | 2.875 |
| **IOA** | 0.566 | 0.529 | 0.674 | 0.815 | 0.809 | 0.665 |
| **Station Name** | **Cooper Hospital** | **D Ward** | **Dadar** | **Dahisar** | **Dindoshi** | **F_N Ward** |
| **RMSE** | 3.163 | 1.800 | 3.791 | 1.023 | 1.613 | 3.280 |
| **PCC** | 0.705 | 0.290 | 0.610 | 0.876 | 0.000 | 0.879 |
| **MAE** | 2.647 | 1.368 | 3.256 | 0.746 | 1.280 | 2.876 |
| **IOA** | 0.603 | 0.389 | 0.572 | 0.867 | 0.000 | 0.623 |
| **Station Name** | **F_S Ward** | **Frosberry Pumping** | **G_S Ward** | **Gawanpada** | **Grant Road** | **HTT** |
| **RMSE** | 2.835 | 2.391 | 4.108 | 0.907 | 4.684 | 3.028 |
| **PCC** | 0.611 | 0.837 | 0.735 | 0.781 | 0.825 | 0.698 |
| **MAE** | 2.072 | 1.752 | 3.443 | 0.676 | 4.145 | 2.520 |
| **IOA** | 0.628 | 0.698 | 0.573 | 0.858 | 0.556 | 0.617 |
| **Station Name** | **H_E Ward** | **H_W Ward** | **Haji Ali Pumping** | **K_E Ward** | **K_W Ward** | **Kandivali** |
| **RMSE** | 4.125 | 4.397 | 5.228 | 1.153 | 3.050 | 7.104 |
| **PCC** | 0.896 | 0.783 | 0.774 | 0.874 | 0.867 | 0.669 |
| **MAE** | 3.448 | 3.524 | 4.508 | 0.933 | 2.665 | 6.308 |
| **IOA** | 0.597 | 0.576 | 0.594 | 0.838 | 0.626 | 0.483 |
| **Station Name** | **Kandiwali Workshop** | **Kurla** | **L Ward** | **MCGM** | **M_E Ward** | **M_W Ward** |
| **RMSE** | 1.776 | 1.086 | 3.736 | 3.186 | 2.956 | 3.938 |
| **PCC** | 0.000 | 0.898 | 0.856 | 0.702 | 0.594 | 0.788 |
| **MAE** | 1.325 | 0.868 | 3.228 | 2.475 | 2.083 | 3.248 |
| **IOA** | 0.000 | 0.845 | 0.576 | 0.596 | 0.609 | 0.563 |
| **Station Name** | **Malad** | **Malbar Hill** | **Malvani** | **Mandavi** | **Marol** | **Memonwada** |
| **RMSE** | 7.029 | 1.701 | 1.739 | 2.972 | 1.439 | 2.172 |
| **PCC** | 0.640 | 0.674 | 0.834 | 0.643 | 0.745 | 0.752 |
| **MAE** | 6.131 | 1.304 | 1.349 | 2.027 | 1.134 | 1.696 |
| **IOA** | 0.515 | 0.780 | 0.782 | 0.604 | 0.727 | 0.692 |
| **Station Name** | **Mulund** | **N Ward** | **Nariman** | **Rawali** | **S Ward** | **SWD** |
| **RMSE** | 1.923 | 3.444 | 1.452 | 4.509 | 1.692 | 4.952 |
| **PCC** | 0.000 | 0.770 | 0.755 | 0.635 | 0.863 | 0.801 |
| **MAE** | 1.412 | 2.759 | 1.176 | 3.416 | 1.252 | 4.222 |
| **IOA** | 0.000 | 0.587 | 0.846 | 0.539 | 0.763 | 0.549 |
| **Station Name** | **SWM Santaceuz** | **Versova Pumping** | **Vikhroli** | **Ville Parle** | **Wadala** | **Worli** |
| **RMSE** | 0.890 | 1.772 | 1.229 | 0.957 | 1.361 | 3.429 |
| **PCC** | 0.893 | 0.000 | 0.800 | 0.849 | 0.782 | 0.846 |
| **MAE** | 0.736 | 1.374 | 0.923 | 0.733 | 0.950 | 2.708 |
| **IOA** | 0.912 | 0.000 | 0.838 | 0.886 | 0.826 | 0.627 |
